# Metal-Dependent Mechanism of the Electrocatalytic Reduction of CO_2_ by Bipyridine Complexes Bearing Pendant Amines: A DFT Study

**DOI:** 10.1021/acsorginorgau.4c00046

**Published:** 2024-10-11

**Authors:** Mahika Luthra, Abril C. Castro, David Balcells, Kim Daasbjerg, Ainara Nova

**Affiliations:** † Hylleraas Centre for Quantum Molecular Sciences, Department of Chemistry, University of Oslo, Oslo 0315, Norway; ‡ Center for Materials Science and Nanotechnology, Department of Chemistry, University of Oslo, Oslo 0315, Norway; § Novo Nordisk Foundation (NNF) CO_2_ Research Center, Interdisciplinary Nanoscience Center, Department of Chemistry, Aarhus University, Gustav Wieds Vej 10C, Aarhus C 8000, Denmark

**Keywords:** carbon dioxide reduction, electrocatalysis, reaction mechanism, computational study, rhenium, ruthenium, molecular complex

## Abstract

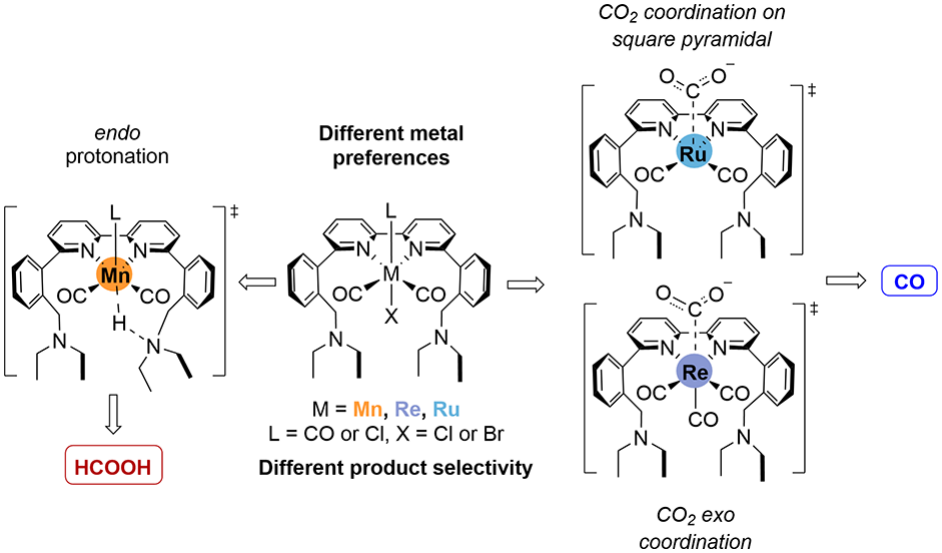

In this study, the electrocatalytic reduction of carbon dioxide by Mn^I^, Re^I^, and Ru^II^ bipyridine complexes bearing pendant amines is evaluated by DFT methods. Prior experimental studies showed that introducing pendant amines in the secondary coordination sphere of the catalyst shifts product selectivity from CO to HCOO^–^ (in the presence of a proton source) in the case of Mn. In contrast, CO is the major product with Re and Ru. This work includes a comprehensive study of the pathways leading to CO, HCOO^–^, and H_2_ to elucidate the energetic preferences that underlie product selectivity. Our results show that switching the metal center leads to changes in the preferred mechanism. While with Mn, the reaction is preferred in an *endo* configuration, allowing the participation of amines in the hydride formation, reactivity on the *exo* configuration is preferred with Re. In addition, the distinct redox properties of Re allow for the formation of Re OCOCO_2_-bridged adducts that lead to CO without a proton source. Further, the ability of Ru to exchange the two Cl^–^ anions changes the preferred coordination number of Ru compared to Mn and Re and, consequently, its reaction mechanism. Overall, this study provides the structure and reactivity insight needed for further catalyst design.

## Introduction

Addressing global warming necessitates exploring sustainable energy sources and viable carbon alternatives capable of replacing fossil fuels. Central to this pursuit is the critical need to avert the deleterious effects of greenhouse gas emissions, particularly CO_2_, by harnessing renewable energies like solar, wind, and waterpower.^1,2^ This transformation can be enabled by the CO_2_ reduction reaction (CO_2_RR), where an electrocatalyst is used to overcome the activation barrier associated with the inherent stability of CO_2_. Metal complexes are interesting candidates as molecular electrocatalysts due to their well-defined structures allowing accurate optimization of the catalytic center. The main challenge of electrocatalysis is to obtain high turnover rates with reasonable overpotential and achieve high selectivity toward a single product. Thermodynamic factors, such as hydricity^3^ and *pK*_a_,^4^ are important descriptors that affect product selectivity. However, selectivity is also influenced by kinetics, for which information on transition states is needed through calculations or experiments.

**Scheme 1:**
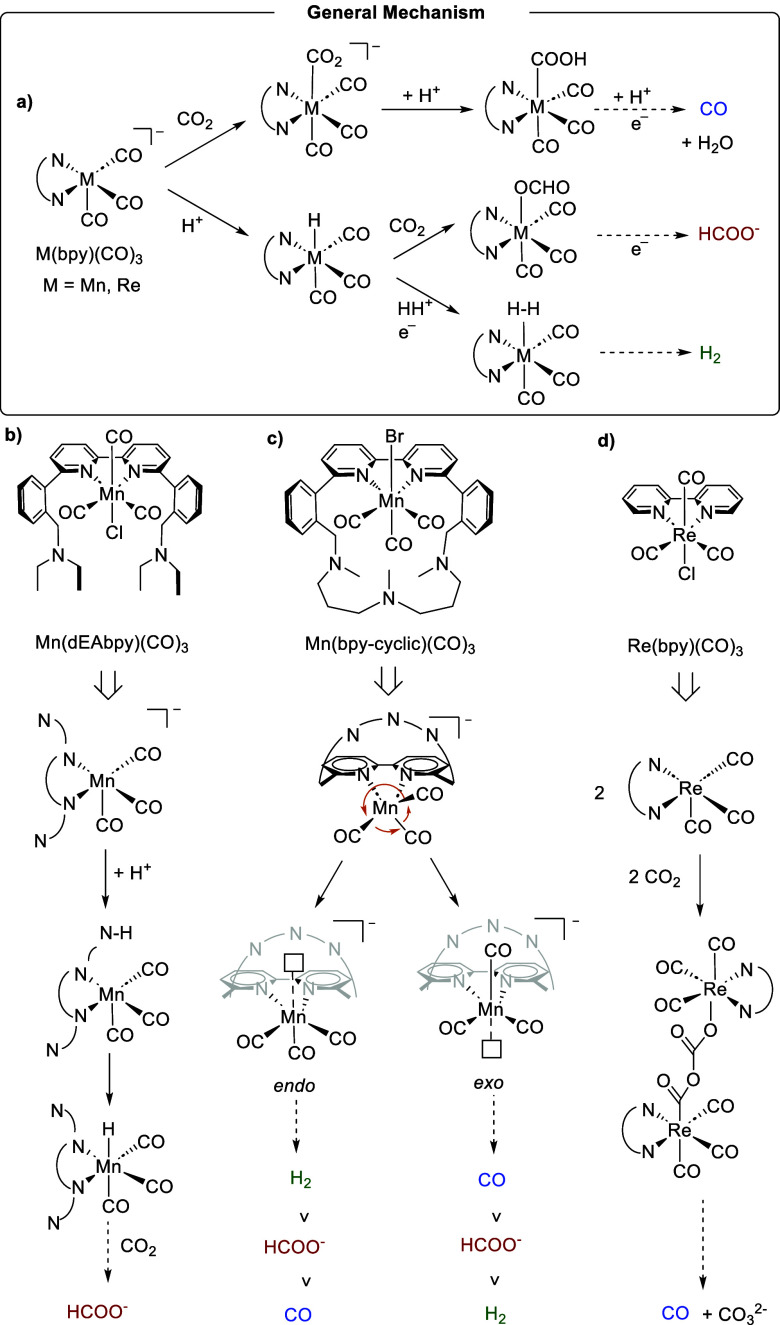
DFT Supported Mechanisms for the Electrocatalytic Reduction of CO_2_ Yielding CO, HCOO^–^, and H_2_ via M(bpy)(CO)_3_ Type Catalysts (a) with Details for Mn dEAbpy (b) and bpy-Cyclic (c) Ligands, and Re bpy (d)

Among the molecular catalysts, *fac*-M^I^(bpy)(CO)_3_X (M = Mn, Re; X = Cl, Br) are well-known for their ability to reduce CO_2_ electrochemically.^5−11^ The fundamental mechanisms of these species are well established, involving two key intermediates, i.e., the metallocarboxylic acid (M-COOH), which leads to CO formation,^5,12^ and metal-hydride intermediate (M-H), resulting in the formation of HCOO^–^.^13^ A competitive hydrogen-evolution reaction can also occur from the metal-hydride intermediate (Scheme 1a).^3^

In a theoretical study by Kubiak et al., a comparison between Mn and Re metal centers in M(bpy)(CO)_3_ catalysts was performed. They showed that the CO_2_ addition was endergonic for the Mn complex and exergonic for the Re complex. Moreover, they found that the hydride formation was exergonic for both.^12^ Additional computational studies were performed on such catalysts with different substituents on the bpy ligand.^12,14−18^ In all these cases, the major CO_2_ reduction product was CO, followed by HCOO^–^ and H_2_. This occurred due to the higher energy barrier for the protonation of the metal to form M-H compared to CO_2_ addition to the metal center to form M-COOH, favoring the formation of CO.^12^

Attempts to increase HCOO^–^ formation by using external tertiary amines, allowed to switch the product selectivity from CO to HCOO^–^ by promoting M-H formation.^16^ Furthermore, in a recent study, the selectivity could be increased toward formate by placing the amines in the ligand in an open configuration (Scheme 1b).^19^ However, in a cyclic configuration, an increased selectivity toward H_2_ was observed experimentally (Scheme 1c). A computational study on this system, performed by our group, showed that the ligand hinders the addition of CO_2_ to the M-H moiety and favors the intramolecular protonation of the hydride, leading to the dominant formation of H_2_.^20^ Both low-overpotential (protonation first) and high-overpotential (reduction first) pathways^21^ were considered for studying the mechanism of CO_2_ reduction. Additionally, ab initio molecular dynamics (AIMD) simulations showed a free rotation of the Mn(CO)_3_ moiety, which generates two conformers, one with the vacant site facing the pendant amines, named *endo*, and the other with the vacant site facing opposite to the pendant amines, named *exo*. This observation showed that the amines could assist the hydride formation even though they were not initially positioned on the halide side. In contrast to Mn, a bimetallic mechanism has been proposed based on DFT calculations for Re(bpy)(CO)_3_ complex in the absence of amine. In this reaction, two singly reduced Re complexes form a bridging CO_2_ adduct followed by CO and CO_3_^2–^ release as shown in Scheme 1d.^22^

**Scheme 2:**
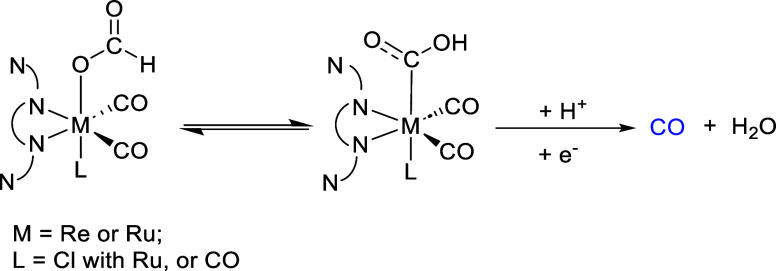
(a) Electrocatalysts with Pendant Amines in an Open configuration; (b) Mechanistic Proposal for the Interconversion of M-OCHO to M-COOH, Consistent with Higher CO Yields; *Reproduced from*;^23^*Copyright 2020 American Chemical Society*

The change in selectivity observed using amines in M(bpy)(CO)_3_ systems with M = Mn has not been experimentally observed with Re and Ru using pendant amines (Scheme 2).^23^ The major reaction product for Re and Ru catalysts was CO, with a Faradaic efficiency (FE) higher than 50%, mixed with smaller amounts of HCOOH (FE = 11.8% for Re and 8.7% for Ru) and H_2_ (FE < 2% for Re and 3–6% for Ru).

For the Re complex, IR studies indicated that despite the presence of pendant amines, there was no direct evidence of the formation of a Re–H species, which is a key intermediate for the formation of HCOO^–^. Moreover, this complex showed CO_2_ reactivity even in the absence of an external proton source, which was essential for Mn complex, which requires a proton source to show CO_2_ reactivity. In the case of the Ru complex, the presence of the Ru–H intermediate was detected, suggesting a potential pathway involving the formato intermediates during the catalytic process. Although no DFT calculations were performed to elucidate the mechanism, it was proposed that once Ru–H is formed and CO_2_ is inserted to form Ru–OCHO, there would be an isomerization of the Ru–OCHO intermediate into the Ru–COOH (Scheme 2b). This mechanism, previously proposed for the Ru complex without pendant amines,^24^ could explain the high amounts of CO observed experimentally (Scheme 2). Moreover, the transition state for such interconversion has been obtained by DFT calculations with Fe and Co-based electrocatalysts.^25^

In this study, we aim to address the following issues arising from prior research efforts: (a) identifying the key parameters influencing the product selectivity of CO_2_ reduction reactions upon switching the metal center from Mn to Re and Ru, with the pendant amines in an open configuration (Scheme 2a), (b) investigating the occurrence of M(CO)_3_ moiety rotation to generate *endo* and *exo* conformers as observed in the case of cyclic amines, and (c) assessing potential mechanistic disparities between Re and Ru catalysts compared to Mn. To tackle these questions, we employ DFT methods. In addition, AIMD simulations are utilized to analyze the dynamic behavior of active species. The DFT-derived energy profiles are used for comparing product pathways for CO_2_ reduction, specifically CO, HCOO^–^, and H_2_. We also explore alternative pathways, such as the bimetallic mechanism and the interconversion of M-OCHO to M-COOH in the presence of pendant amines. Interestingly, varying the metal center prompts distinct mechanisms among these catalysts, thereby influencing product selectivity. This elucidation aids in explaining observed experimental variations in product selectivity.

## Methods

Density functional theory with the solvation model based on density (DFT/SMD)^26^ and AIMD^27^ were used in this study. The DFT/SMD calculations were performed using the Gaussian 16 C.01^28^ software with the TPSSh-D3 functional and acetonitrile as solvent.^29^ The def2SVP^30^ basis set was used for geometry optimization, and def2TZVP^31^ (Mn, Re and Ru) and def2TZVPD^31^ (remaining atoms) for energy refinement. The AIMD simulations were performed using the CP2K program package^32^ with the PBE functional^33^ and a mixed DZVP Gaussian and auxiliary plane wave (200 Ry cutoff) basis set, in explicit solvent (acetonitrile).^34^Scheme S1 shows how the two methods have been combined in this study. Other computational details are provided in the Supporting Information. DFT/SMD calculations are performed to optimize the geometries of the initial intermediate 1_MX_ (M = Mn, Re, X = Br, Cl) and derive the active anionic intermediate 2^–^_M_ after a two-electron reduction and halide dissociation (Scheme S1). AIMD simulations of Mn and Re complexes were conducted on the active intermediates 2^–^_M_ and their protonated forms 3_M_. The initial models for AIMD simulations were created with PACKMOL,^35^^,^ surrounding the complexes with 50 CH_3_CN molecules for 2^–^_Mn_ and 25 for 2^–^_Re_, maintaining a density of 0.786 g mL^–1^ (Figure S2). These initial models were relaxed in a microcanonical ensemble to reach 298 K, followed by a canonical ensemble simulation at the same temperature maintained with a CSVR algorithm.^36^ Core electrons were described using pseudopotentials of the Goedecker–Teter–Hutter type.^37^ The trajectories were extended to 25 ps with a 0.25 fs time step, and the lowest energy conformers were extracted and optimized using DFT/SMD calculations (Figures S3 and S4). The reaction mechanism leading to the three products were studied using DFT/SMD. In Schemes 5 and 7, the term “low overpotential pathway” refers to the pathway that only requires the reduction of the initial complex, while the “high overpotential pathway” involves the reduction of one of the reaction intermediates from the “low overpotential pathway” (see Supporting Information for further details).

## Results and Discussion

### Catalyst Activation

**Scheme 3:**
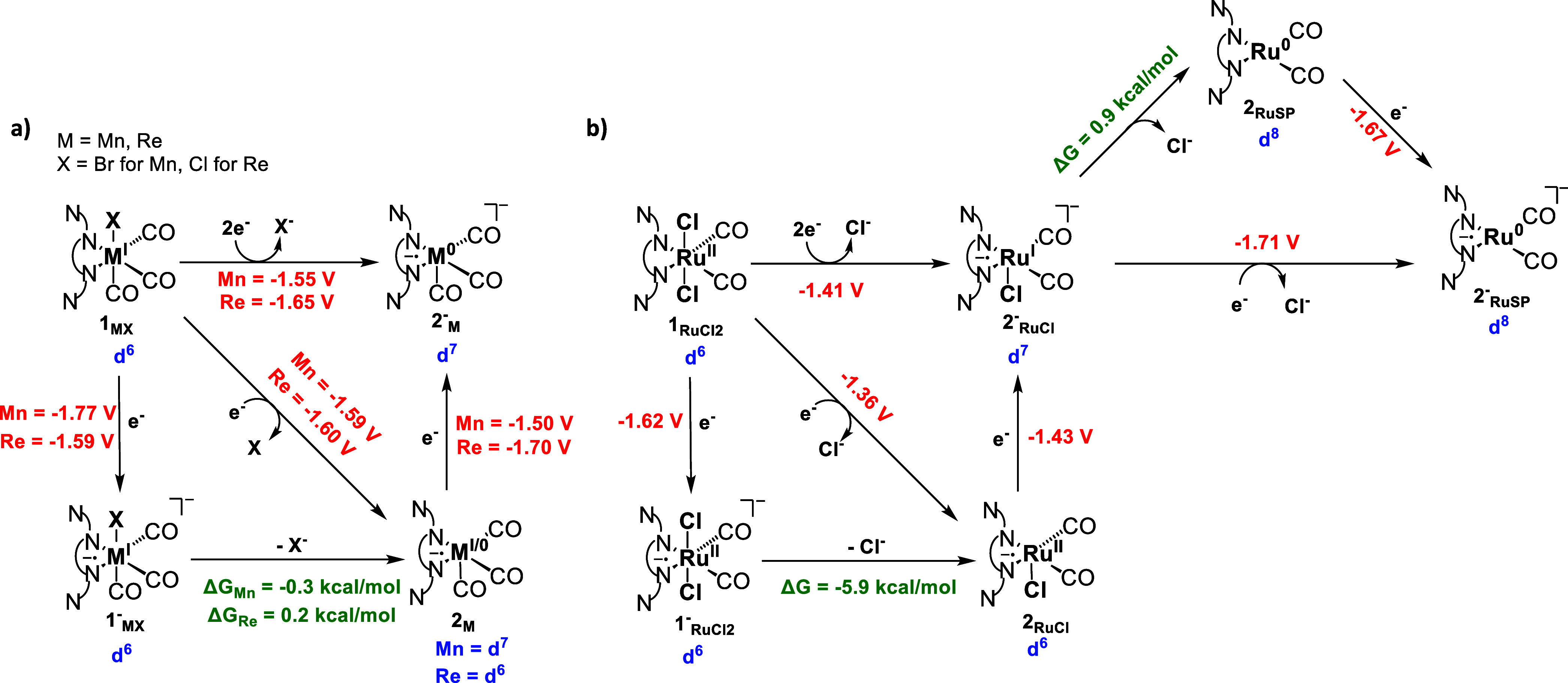
Redox Events for the Activation of the Metal Complexes 1_M_, Where M = (a) Mn, Re, and (b) Ru with the d Electron Count for Each Metal (in Blue), Redox Potentials (in Red) and Free Energies for the Thermodynamic Events (in Green) Redox potentials are calculated relative to the Fc^+^/Fc couple.

The activation process of the catalysts with open ligand design is similar to the one with the cyclic ligand.^20^ It starts from its initial state 1_MX_ (M = Mn, Re, Ru), where a halide (X = Br or Cl) is attached to the central metal atom. Following a two-electron reduction event, the resulting anionic complex (2^–^_M_) is the pivotal starting point for subsequent CO_2_ reactivity. This two-electron reduction can be either a one- or two-step process. In the one-step process, a direct two-electron reduction from 1_MX_ to 2^–^_M_ occurs, transitioning the catalyst from its initial state to the anionic complex. Conversely, the two-step process involves two one-electron redox events: 1_MX_ → 2_M_ and 2_M_ → 2^–^_M_ (Scheme 3), resulting in the formation of the same anionic intermediate. It is essential to note that in the case of Mn and Re, the initial structures (1_MnBr_ and 1_ReCl_) featured three CO ligands and one halide, whereas Ru (1_RuCl2_) featured two CO ligands in *cis* and two chlorides in *trans* occupying the axial positions.

Although previously reported,^19^ the activation mechanism for 1_MnBr_ was computed using the same methodology used for Re and Ru for comparison. The redox potential values show that the direct two-electron reduction of the initial catalyst 1_MnBr_ → 2^–^_Mn_ (Scheme 3a) was favored in both our case and the previous study, with redox potential values of −1.55 and −1.50 V, respectively. These values corresponded well with the experimental redox potential of −1.55 V.

In the case of 1_ReCl_ (Scheme 3a), we observed that the initial reduction, following a two-step process (1_ReCl_ → 1^–^_ReCl_ → 2_Re_), had a redox potential of −1.59 V, where the Cl^–^ ion dissociation to form 2_Re_ is slightly endergonic by 0.2 kcal mol^–1^. Interestingly, if we consider a one-step process where the reduction and chloride removal occur simultaneously (1_ReCl_ → 2_Re_), the redox potential remains similar at −1.60 V, aligning with the two-step process. Once 2_Re_ is formed, a second reduction (2_Re_ → 2^–^_Re_) occurs with a higher redox potential of −1.70 V, compared to the first electron reduction. Even when examining a direct two-electron reduction (1_ReCl_ → 2^–^_Re_), the redox potential remains higher at −1.65 V compared to the first reduction. This indicates that the formation of 2_Re_ is energetically more favorable than 2^–^_Re_. An analysis of natural charges and spin density in the Re complex (Table S4) revealed that after the initial reduction, the electron was localized in the bpy ligand, even after the chloride detachment. During the second reduction (2_Re_ → 2^–^_Re_), the incoming electron became centered on the Re metal, in line with a d^7^ electronic configuration. It is also important to note that the doubly reduced species 2^–^_Re_ and 2^–^_Mn_ are both closed shell singlets (Tables S3 and S4), even though they are formally assigned a M(0) d^7^ electron configuration. This result agrees with previous DFT calculations and molecular orbital analysis for analogous systems without pendant amines.^12,38^ Overall, the pathway followed by the Re complex is a fast 1_ReCl_ → 2_Re_ first reduction followed by a slow 2_Re_ → 2^–^_Re_ second reduction, which is also consistent with experimental observations, where two different reduction waves are observed, with potentials of −1.82 and −2.3 V. This behavior differs from the one observed with Mn, in which the formation of the anionic intermediate 2^–^_Mn_ was found to be more favorable energetically than the formation of 2_Mn_. Additionally, in 2_Mn_, the electron is transferred to the metal. This difference is likely attributed to the higher crystal field splitting of heavy transition metals compared to first-row transition metals, potentially explaining why the transition from d^6^ to d^7^ is more facile with Mn than Re. Moreover, the deviation of the computed redox potentials from the experimental values is more pronounced in the case of Re, as compared to Mn. This can be explained by relativistic effects, which are more pronounced in heavier elements.

The behavior of Ru complex, 1_RuCl2,_ deviates from that of Mn and Re due to its placement in group 8 of the periodic table, in contrast to Mn and Re, which belong to group 7 (Scheme 3b). As a result, the initial oxidation state of Ru is +2 and has two axial chlorides that need to be removed through two cycles of reduction, with the first cycle resulting in the formation of 2^–^_RuCl_ and the second cycle forming 2^–^_RuSP_ (SP stands for square planar). In the first cycle, the reduction 1_RuCl2_ → 1^–^_RuCl2_ takes place with the redox potential of −1.62 V. Following this reduction, chloride release is exergonic by 5.9 kcal mol^–1^, resulting in the formation of 2_RuCl._ The direct reduction of 1_RuCl2_ to 2_RuCl_ has a redox potential of −1.36 V. A second electron addition to form 2^–^_RuCl_ takes place at a redox potential of −1.43 V. The higher potential for the second reduction than the first indicates the preference for forming 2_RuCl_. A direct 2-electron reduction from 1_RuCl2_ to 2^–^_RuCl_ is also calculated to be −1.41 V, which shows the most convenient way to form 2^–^_RuCl_ from 1_RuCl2_. Moreover, because of the +2 oxidation state, the overall redox potential values for Ru are significantly lower than Re. In the second cycle, the second chloride is removed by a single electron reduction with the redox potential of −1.71 V to form 2^–^_RuSP_. The examination of the natural charges and spin densities (Tables S5 and S6) reveals that the formal oxidation state of Ru is zero in 2^–^_RuSP_ with a d^8^ electronic configuration, which favors the formation of a square planar complex. The computed redox potentials are in qualitative agreement with the experimental observation, which reveals two prominent reduction waves, with the first being twice as high as the second, with redox potentials of −1.63 and −2.07 V.

### Dynamic Behavior

After achieving catalyst activation to produce the active intermediate 2^–^_M_, AIMD simulations were employed to investigate its flexibility. Additionally, the impact of protonation on this intermediate, named 3_M_, was explored using the same approach applied to the Mn cyclic system in Scheme 1c.^20^ Due to the different coordination geometries of 2^–^_M_ complexes with Mn and Re (square pyramidal) and Ru (square planar), AIMD simulations were exclusively performed for Mn and Re.

**Figure 1:**
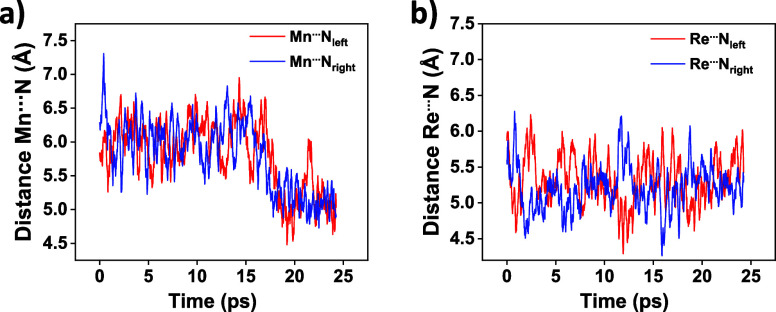
Time evolution of the M···N_left_ (red) and M···N_right_ (blue) distances for the MD simulation trajectory of the (a) 2^–^_Mn_ and (b) 2^–^_Re_ intermediates.

The trajectories of the intermediates 2^–^_Mn_ and 2^–^_Re_ revealed the rotation of the CO ligands around the metal center, similar to the cyclic complex with Mn,^20^ generating *endo* and *exo* conformers. To further elucidate the interaction between the vacant site and the amines in 2^–^_M_ complexes, we monitored the distances between the metal and the nitrogen atoms of the pendant amines (M···N_left_ and M···N_right_), as shown in Figure 1 and Table S9. The average distances between the M and N atoms were similar for both pendant amines (5.81 and 5.80 Å for Mn; 5.31 and 5.30 Å for Re), indicating the symmetric behavior of the ligand. This differs from the cyclic complex, where the Mn···N distances for the three pendant amines varied notably.^20^

**Figure 2:**
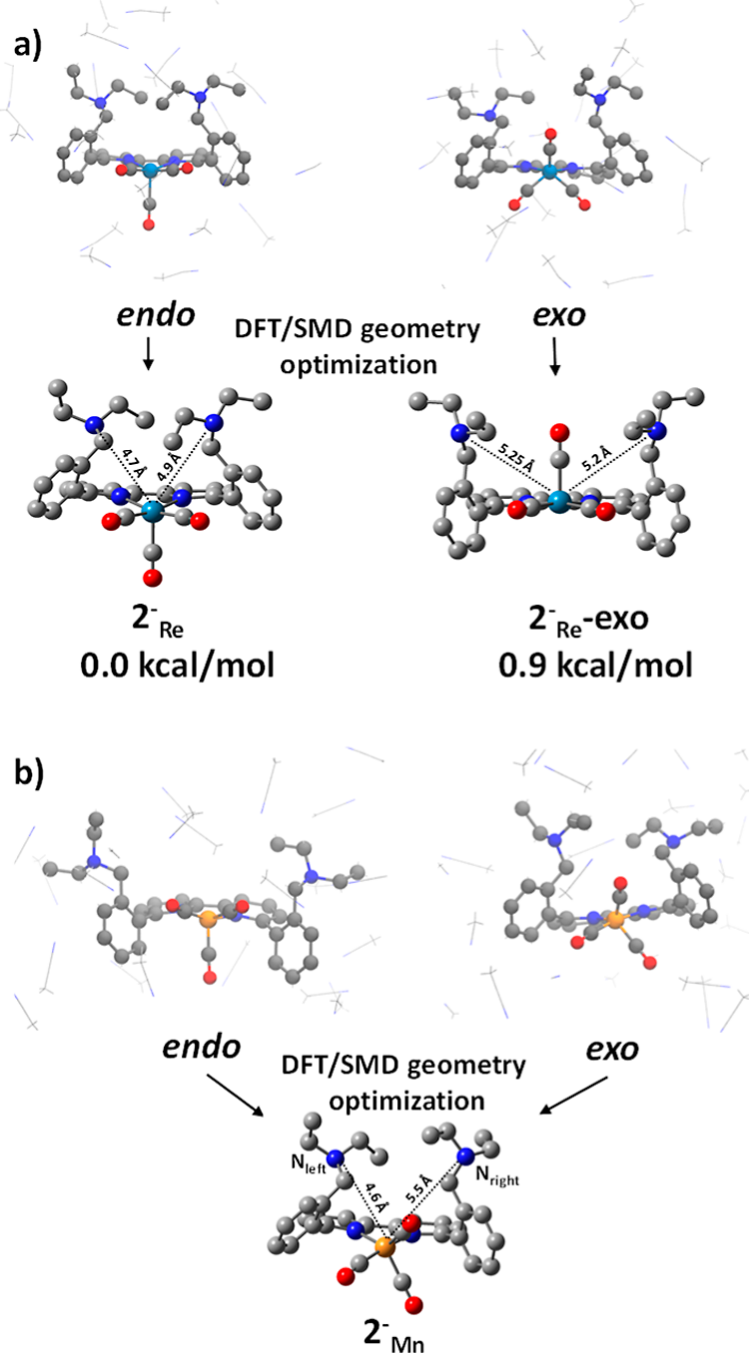
Isolation of *endo* and *exo* conformers from the MD simulations followed by DFT/SMD geometry optimization for the 2^–^_M_ complex, where M = (a) Mn, (b) Re.

In the case of 2^–^_Re_, extracting the *endo* and *exo* conformers from the AIMD trajectory resulted in two distinct structures after DFT/SMD optimization (Figure 2a). The average dihedral angle formed between the three CO ligands with Re metal and the atoms N_bpy_ and C_bpy_ of the bpy plane was calculated for the DFT-optimized structures. For the *endo* conformer, it was −55.09°, and for the *exo* conformer, it was 60.79°. These two structures exhibited a small energy difference of 0.9 kcal mol^–1^. Regarding 2^–^_Mn_, both the *endo* and *exo* conformers extracted from the AIMD simulations converged into the same structure when optimized with DFT/SMD (Figure 2b). The vacant site location in this geometry was uncertain, making it challenging to determine whether it should be classified as *endo* or *exo*. Additionally, we performed DFT/SMD calculations for the *endo* and *exo* conformations of 2^–^_Mn_ using PBE functional and def2SVP/def2TZVP basis set. We obtained similar results as with the TPSSh functional, suggesting that the *endo* and *exo* conformations result from a dynamic effect. The average dihedral angle in 2^–^_Mn_ formed by each CO ligand with Mn and the bpy plane was −36.70°. This value resembled the *endo* conformer (−55.09°) more than the *exo* conformer (60.79°) observed for Re. Moreover, it was closer to the structure of the cyclic complex, where the average dihedral angle was −41.09°, indicating a predominant *endo* character.^20^ Additional details on how these conformers are extracted from the MD trajectory are provided in the Supporting Information (Figures S3 and S4).

**Figure 3:**
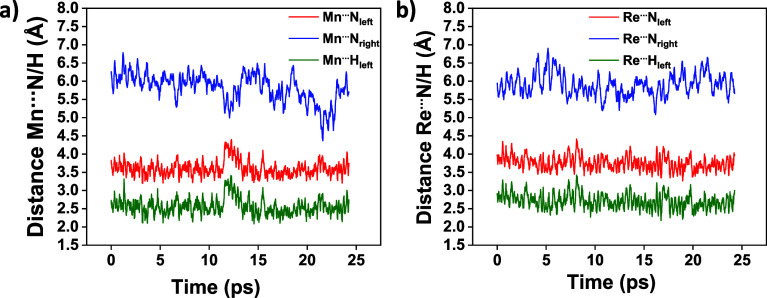
Time evolution of the M···N_left_ (red), M···N_right_ (blue), and M···H_left_ (green) distances along the AIMD trajectory of (a) 3_Mn_ and (b) 3_Re_ intermediates.

To explore the impact of protonation on the M···N distances, we also run AIMD simulations for the complexes with a protonated amine, 3_M_. Figure 3 shows the Mn···N_left_, M···N_right_, and M···H_left_ distances along the 3_M_ trajectories (H represents the proton added on the left amine). The protonated (left) amine exhibited a lower Mn···N_left_ average distance of 3.60 Å in 3_Mn_ (Table S10), compared to 5.81 Å in 2^–^_Mn_ (Table S9). Conversely, the nonprotonated amine, N_right_, remained farther from the metal center, averaging 5.82 Å in distance. This trend is similar to that observed in the cyclic amine, where the protonation of the central amine reduced the average Mn···N_M_ distance, compared to the distances in the nonprotonated amines. Similarly, for 3_Re_, the average Re···N_left_ distance decreased from 5.30 Å in 2^–^_Re_ to 3.75 Å.

Notably, these observations highlight the similarities in the dynamic behavior of both cyclic and open ligand complexes with Mn, as well as those with Re as the metal center. A consistent finding in both complexes is that the protonation of the 2^–^_M_ intermediate facilitates the formation of a metal-hydride complex by decreasing the M···NH distances, thereby marking this step crucial in the catalytic process.

### Mechanistic Investigation

The mechanism for CO_2_ reduction was explored by computing and comparing the energy profiles of the reaction pathways yielding these three products: HCOO^–^, CO, and H_2_, all starting from the active intermediate 2^–^_M_ (M = Mn, Re), which was extracted from the AIMD simulations. For Ru, the mechanism was studied starting from the intermediate 2^–^_RuSP_.

#### Mn Catalyst

Adding to the previous work^19^ on the mechanism of HCOO^–^ formation with complex 1_Mn_, we hereby report the energy profile for the formation of H_2_ and CO with the *endo* and *exo* conformers (Schemes S2–S6). Similar to the cyclic system, the overall pathway toward CO formation exhibited higher energy barriers (TS9_Mn_ – 8_Mn_ = 16.7 kcal mol^–1^) compared to the H_2_/HCOO^–^ pathways (TS2_Mn_ – 3_Mn_ = 9.3 kcal mol^–1^) in the low overpotential pathway. The preference for formate instead of H_2_ is explained by the higher energy barriers for H_2_ formation (TS4_Mn_ – 4_Mn_ = 8.9 kcal mol^–1^) compared to the formation of HCOO^–^ (TS5_Mn_ – 4_Mn_ = 4.9 kcal mol^–1^), in line with the experimental preference for HCOO^–^ formation. This was the opposite in the case of the cyclic system where the formation of HCOO^–^ showed a higher energy barrier of 19.2 kcal mol^–1^ compared to that of H_2_ formation (17.7 kcal mol^–1^), due to steric effects on the addition of CO_2_ to the Mn–H bond.^20^ The energies for the high overpotential pathway also show a preference for HCOO^–^ formation, with a low energy barrier of 3.7 kcal mol^–1^ (5.3 kcal mol^–1^ for H_2_; Scheme S3). The energy profile for the *exo* pathway was also computed and found to be less favorable than the *endo* (Scheme S5). Interestingly, in the *exo* pathway, the formation of CO involves lower energy barriers compared to HCOO^–^ and H_2_ since the pendant amines cannot act as proton shuttles. The *endo* barrier for HCOO^–^ formation (7.7 kcal mol^–1^) is significantly lower than that of CO in the *exo* pathway (19.7 kcal mol^–1^), which makes the *endo* pathway likely to be followed. However, small amounts of CO are observed experimentally because of the competitive barriers to CO and H_2_/HCOO^–^ formation by the low overpotential pathway (TS6_Mn_ and TS2_Mn_, respectively). This leads to the formation of intermediates 9_Mn_ and 4_Mn_, which can be reduced, giving access to the high overpotential pathways.

#### Re Catalyst

**Scheme 4:**
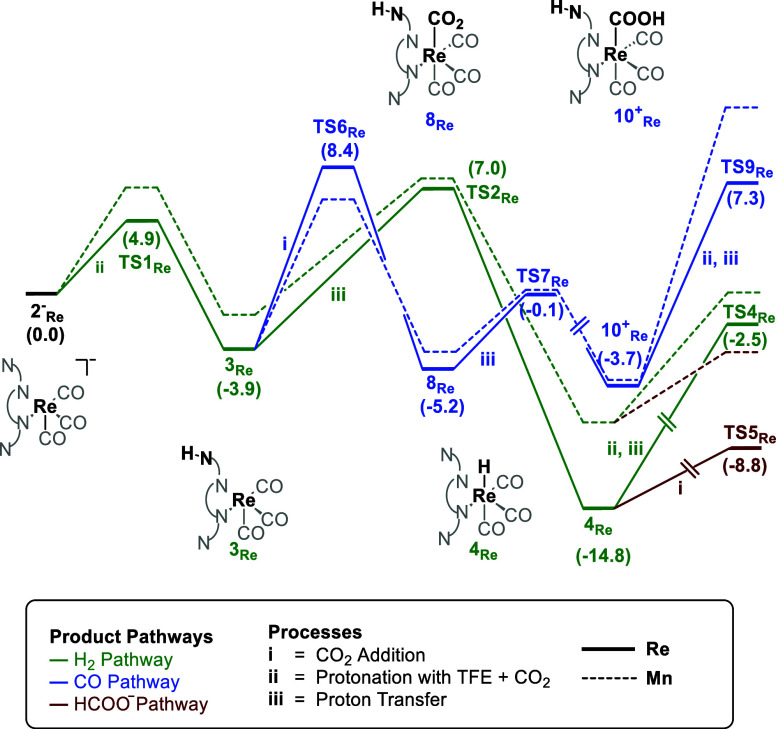
Gibbs Energy Profile (kcal mol^–1^) for the Reduction of CO_2_ with TFE as a Proton Source to H_2_/HCOO^–^ (Green) and CO (Blue) Using Re (Solid Lines) and Mn (Dashed Lines) with the *endo* Isomer The labels and energies are only marked for the Re complex. Some intermediates and TSs have been omitted for clarity.

**Scheme 5:**
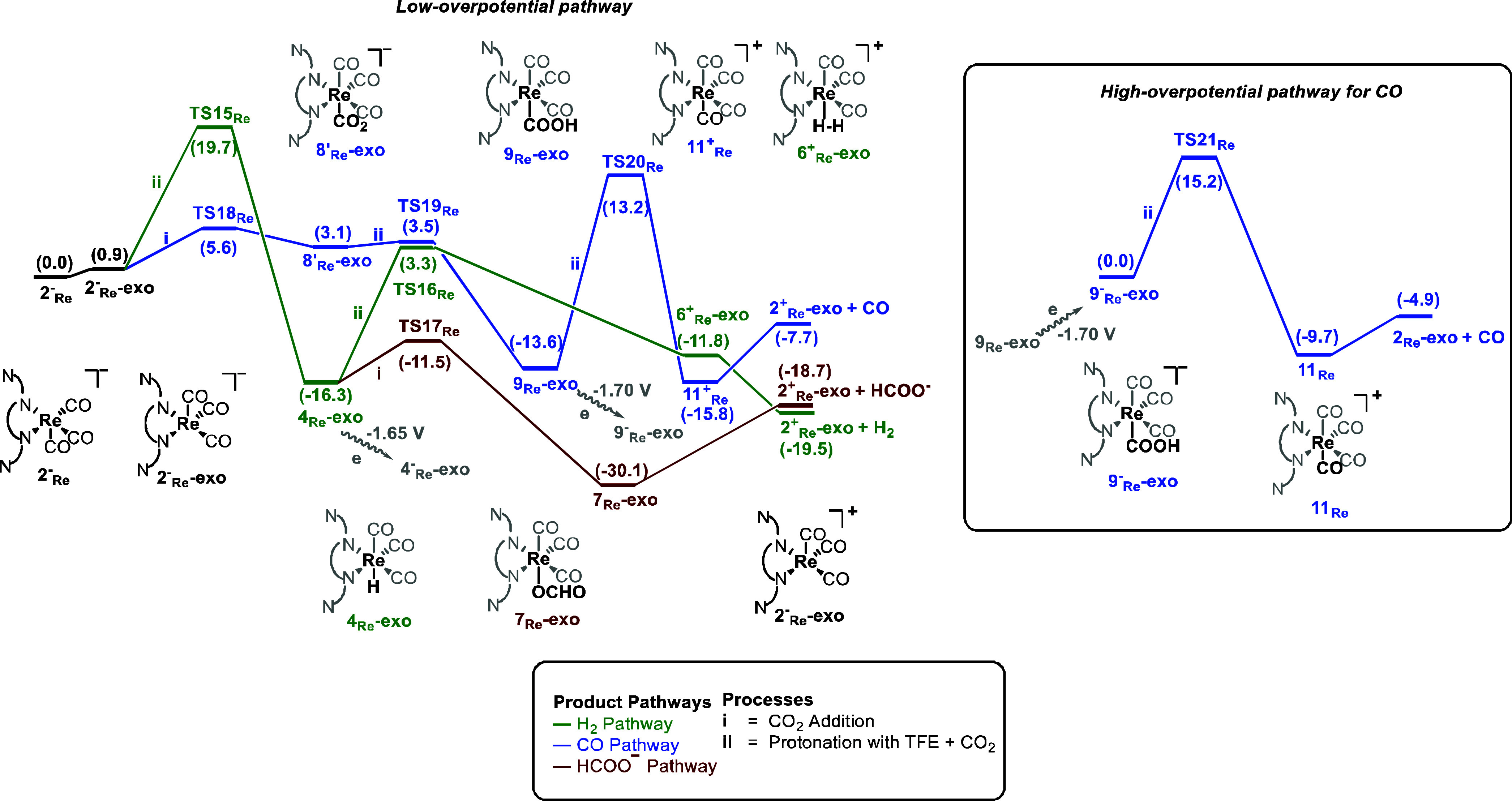
Gibbs Energy Profile (kcal mol^–1^) for the Reduction of CO_2_ with TFE as a Proton Source to Three Different Products: H2 (Green), HCOO^–^ (Red), and CO (Blue) Using the Re Complex with the *exo* Isomer Starting from 2^–^_Re_-exo for the Low-Overpotential Pathway and from 9^–^_Re_-exo for the High-Overpotential Pathway for CO Production Using Trifluoroethanol (TFE) as the Proton Source

**Scheme 6:**
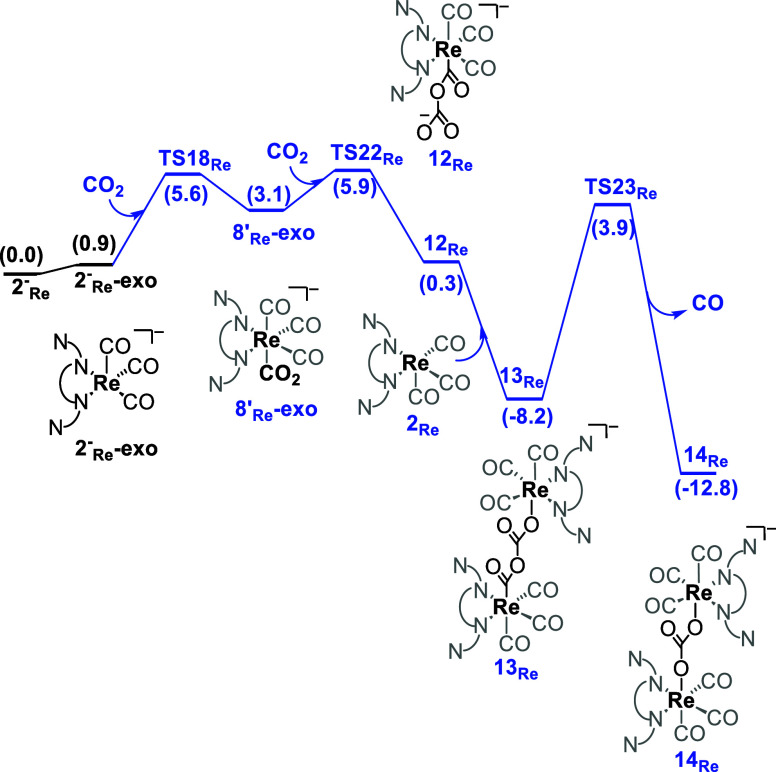
Gibbs Energy Profile (kcal mol^–1^) for the Bimetallic Pathway Starting from 2^–^_Re_-exo to Release CO

The mechanism for the formation of CO, formate and H_2_ was also explored using the Re^I^ catalyst. Scheme S7 shows the complete energy profile diagram starting with the 2^–^_Re_ complex and considering the reaction occurring from the *endo* isomer. When compared with the 1_MnBr_ complex (see Scheme 4 for a simplified version), the energies for the different pathways followed a similar trend; that is, higher energy barriers for forming CO, with the overall energy barrier of 13.0 kcal mol^–1^ (TS9_Re_ – 10^+^_Re_), compared to H_2_ and HCOO^–^, with overall barriers of 12.3 kcal mol^–1^ (TS4_Re_ – 4_Re_, Scheme 4) and 6 kcal mol^–1^ (TS5_Re_ – 4_Re_, Scheme 4), respectively. The formation of formate is also preferred in the high overpotential pathway (Schemes S8 and S9), which is accessible because the experimental applied potential is higher (−2.37 V) than the calculated redox potentials for 4_Re_ and 9_Re_ (−1.64 and −1.73 V, respectively). Comparing these results to previous computational studies on the (bpy)Re(CO)_3_ complex without pendant amines,^5−11^ in which CO formation is preferred, one would suggest that amines also switch product selectivity to formate with the Re complex. However, experimentally CO is still obtained as the major product. Therefore, we moved our analysis to the *exo* isomer (see Scheme 5). As shown by AIMD simulations (*vide supra*), the energy gap between the *endo* and *exo* conformers of the 2^–^_Re_ intermediate was very narrow, 0.9 kcal mol^–1^. This indicates that the reaction with CO_2_ can also take place through the *exo* isomer without involving the amines as shown in Scheme 5. In this case, the formation of the Re-COOH intermediate, 9_Re_-exo, had a lower energy barrier of 5.6 kcal mol^–1^ (TS18_Re_) than that of the Re–H intermediate, 4_Re_-exo, through direct protonation of the metal center, which is 19.7 kcal mol^–1^. Furthermore, the formation of 9_Re_-exo is also kinetically favored over the formation of the hydride intermediate, 4_Re,_ in the *endo* pathway (TS2_Re_ = 7.0 kcal mol^–1^) and 9_Re_-exo can be reduced to 9^–^_Re_-exo at a redox potential of −1.70 V in the high overpotential pathway (Scheme 5). The energy barrier for forming the tetracarbonyl intermediate 11_Re_ in this pathway is 15.2 kcal mol^–1^ (TS21_Re_). Overall, the most favorable pathway followed by the Re complex is the *exo* pathway, which leads to the formation of CO in line with the experimental observations, in contrast with the Mn catalyst.

Additionally, an alternative pathway for CO_2_ reduction was considered, taking into account the previously reported formation of adducts (see Scheme 6).^39^ In this mechanism, we considered the coexistence of the single and double reduced Re intermediates 2_Re_ and 2^–^_Re_, which should be possible given the lower potential required for the first reduction of the catalyst (1_ReCl_ → 2_Re_) compared to the second (2_Re_ → 2^–^_Re_) (Scheme 2). The reaction starts with the doubly reduced intermediate 2^–^_Re_-exo, which adds CO_2_ to form 8′_Re_-exo. Adding another CO_2_ molecule to this intermediate has an energy barrier of 5.9 kcal mol^–1^, forming the 12_Re_ (Scheme 6). The interaction of this intermediate with the singly reduced species, 2_Re_, yields a bimetallic intermediate 13_Re_ with two CO_2_ molecules bridging the two metals. The rearrangement of this intermediate liberating CO has an energy barrier (TS23_Re_ – 13_Re_) of 12.1 kcal mol^–1^ and is exergonic by 4.6 kcal mol^–1^ (14_Re_ – 13_Re_).

This mechanism also fits the experimental observation that the formation of CO does not require a proton source with this Re complex, unlike the analogous Mn complex. Moreover, the overall energy barrier of 12.1 kcal mol^–1^ for forming CO in this pathway is competitive to the energy barrier of 15.2 kcal mol^–1^ observed in the high overpotential *exo* mechanism (TS21_Re_, Scheme 5). Both these pathways lead to the formation of CO, which explains the higher CO yield relative to HCOO^–^ and H_2_ observed in the experiments. An alternative to the bimetallic mechanism is the coordination of the solvent (CH_3_CN) to the single reduced Re complex 2_Re_. However, this reaction was found to be endergonic by 2.0 kcal mol^–1^ (Scheme S19), hence, it was not considered further in our study.

#### Ru Catalyst

**Scheme 7:**
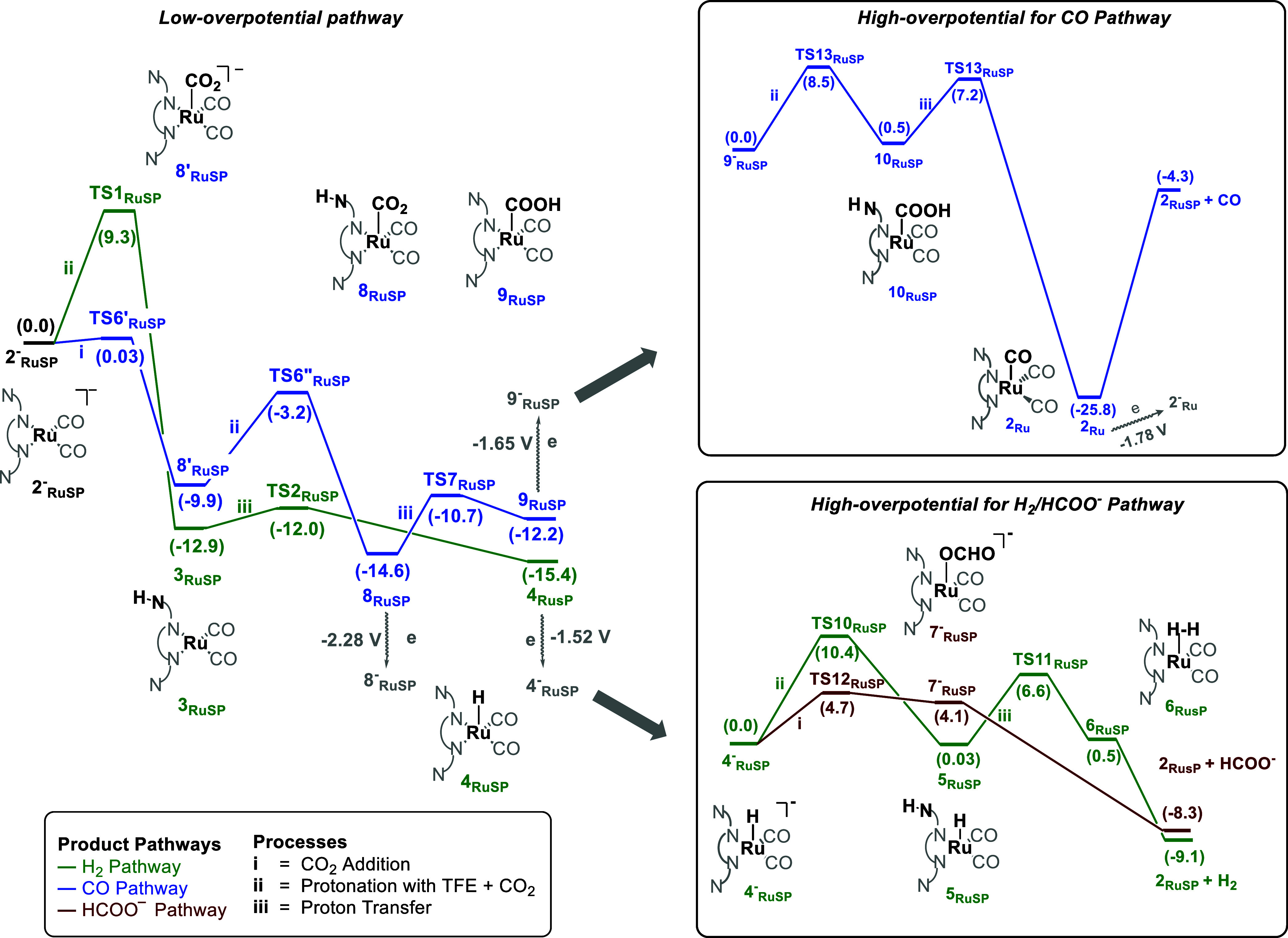
Gibbs Energy Profile (kcal mol^–1^) for the Reduction of CO_2_ with TFE as a Proton Source to Three Different Products: H_2_ (Green), HCOO^–^ (Red), and CO (Blue) Using the Ru Complex from the *endo* Direction Starting from 2^–^_RuSP_ Intermediate for the Low-Overpotential Pathway and from 9^–^_RuSP_ and 4^–^_RuSP_ Intermediates for the High-Overpotential Pathway of CO and H_2_/HCOO^–^ Products, Respectively

The energy profiles for forming formate, CO, and H_2_ were also computed with the reduced tetracoordinated Ru^0^ complex 2^–^_Ru__SP_ (Scheme 7). Unlike the Mn and Re pathways, the energy barrier for the protonation of the amine (TS1_RuSP_ = 9.3 kcal mol^–1^) to form 3_RuSP_ is higher than the energy barrier for CO_2_ addition (TS6′_RuSP_), which has an energy barrier below the diffusion limit of 4.5 kcal mol^–1^.^40^ Still, there is a thermodynamic preference for forming 3_RuSP_ (−12.9 kcal mol^–1^) over 8′_RuSP_ (−9.9 kcal mol^–1^). Following the CO pathway, the Ru–COOH intermediate (9_RuSP_) forms after the protonation and proton transfer steps, with an energy barrier of 3.9 kcal mol^–1^ (TS7_RuSP_ – 8_RuSP_). Following this process, a barrier of 22.5 kcal mol^–1^ (TS9_RuSP_ – 8_RuSP_) is required to liberate CO, while the liberation of H_2_ and HCOO^–^ involve barriers of 14.7 kcal mol^–1^ (TS4_RuSP_ – 4_RuSP_) and 9.3 kcal mol^–1^ (TS5_RuSP_ – 4_RuSP_), respectively. However, the formation of the key 9_RuSP_ intermediate in the CO pathway is still kinetically preferred over that of 4_RuSP_ in the H_2_ and HCOO^–^ pathways. These intermediates can be reduced with redox potentials of −1.65 V (9_RuSP_ → 9^–^_RuSP_) and −1.52 V (4_RuSP_ → 4^–^_RuSP_), which are lower than the experimental applied potential of −2.37 V,^23^ thus giving access to high overpotential pathways.

**Scheme 8:**
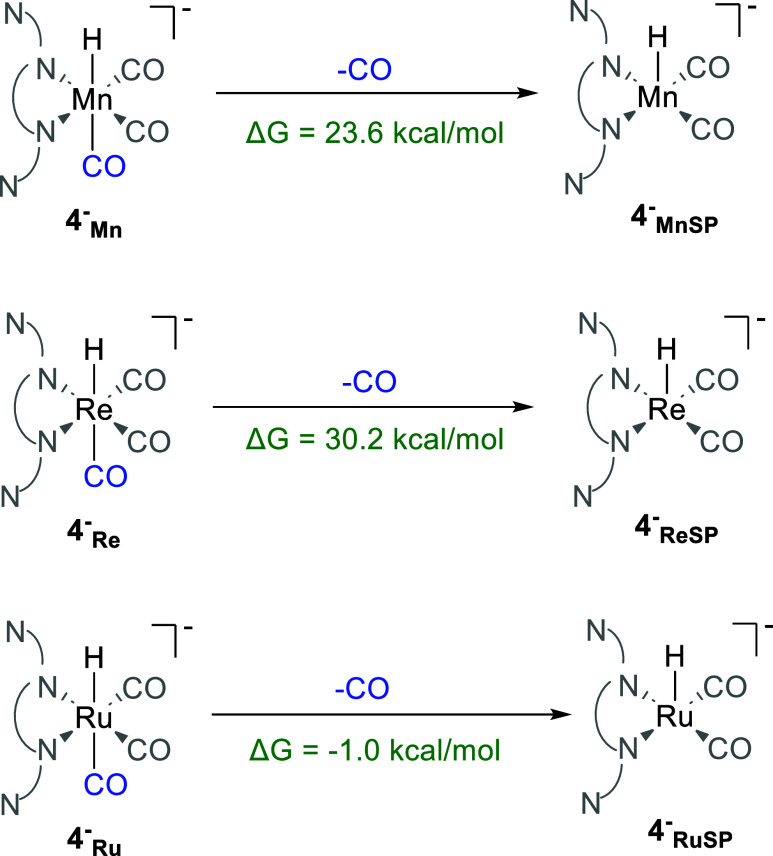
Representation of the CO Dissociation Reactions from 4^–^_Mn_, 4^–^_Re_, and 4^–^_Ru_ Intermediates The energies are thermodynamic free energies (kcal mol^–1^).

Scheme 8 shows the high potential pathway starting from 9^–^_RuSP_. The overall energy barrier to form the tricarbonyl intermediate 2_Ru_ is 8.5 kcal mol^–1^. The formation of 2_Ru_ is exergonic by 25.8 kcal mol^–1^ and irreversible. Subsequently, CO is released to form 2_RuSP_ with an energy cost of 21.5 kcal mol^–1^, which is feasible at room temperature. Scheme 8A explores a high overpotential pathway starting from 4^–^_RuSP_, where the liberation of H_2_ has an overall energy barrier of 10.4 kcal mol^–1^ (TS10_RuSP_). In contrast, the formation of HCOO^–^ involves a lower energy barrier of 4.7 kcal mol^–1^ (TS12_RuSP_), suggesting that HCOO^–^ is more likely to be formed than H_2_. However, the overall selectivity should favor CO, as forming 9_RuSP_ is kinetically more favorable than forming 4_RuSP_. This aligns with the experimental selectivity toward CO, with minor amounts of HCOO^–^, and H_2_.^21^ The energy profiles considering the reaction occurring on the opposite side of the amines (*exo* configuration) were also computed (Scheme S10) and showed a preference for CO with a slightly higher energy barrier.

In the energy profiles yielding CO in the high overpotential pathways (Scheme 7), the formation of the tricarbonyl intermediate, 2^–^_Ru,_ was exergonic and irreversible. Hence, the reactivity of 2^–^_Ru_ was also explored for forming HCOO^–^, H_2_, and CO, similar to the case of 2^–^_Mn_ and 2^–^_Re_ (Schemes S12–S14). The energy profiles show that the barriers for forming CO (TS9_Ru_ – 3_Ru_ = 32.0 kcal mol^–1^) are higher compared to those for H_2_ (TS4_Ru_ – 4_Ru_ = 19.5 kcal mol^–1^) and HCOO^–^ (TS5_Ru_ – 4_Ru_ = 13.5 kcal mol^–1^). Further, the same trend is observed for the energy barriers yielding the key intermediates, Ru–H (4_Ru_) and Ru–COOH (9_Ru_), which are 4.9 kcal mol^–1^ (TS2_Ru_ – 3_Ru_) and 8.2 kcal mol^–1^ (TS6_Ru_ – 3_Ru_), respectively. However, when comparing the redox potentials required to reduce these intermediates, we observed intriguing differences between Ru, Mn, and Re. For Mn and Re, it is less energy-demanding to reduce M-H to M-H(−) (M = Mn, Re) than to reduce M-COOH to M-COOH(−) (Schemes S2 and S7). In contrast, Ru exhibits a reverse trend, being notably easier to reduce Ru–COOH (9_Ru_) to Ru–COOH(−) (9^–^_Ru_), with a potential of −1.78 V, than to reduce Ru–H (4Ru) to Ru–H(−) (4^–^_Ru_), with a potential of −1.96 V (Scheme 7). Further, once 4^–^_Ru_ is formed, the dissociation of the axial CO ligand, which has a Ru···CO(axial) distance of 2.41 Å (Figure S7), forming the pentacoordinate Ru–H (4^–^_RuSP_), is exergonic by −1.0 kcal mol^–1^ (Scheme 8). This is unlike Mn and Re, where the M···CO(axial) distance (Figure S7) is shorter and the CO dissociation is endergonic (Scheme 8). This distinct behavior of Ru may arise from the different electronic configuration of Ru in 4^–^_Ru_ (d^7^) relative to that of Mn and Re in 4^–^_Mn_ and 4^–^_Re_ (d^6^). Following the dissociation of CO in 4^–^_Ru_, the initial Ru(0) square planar structure (2_RuSP_) is recovered by the further reduction of CO_2_ by Ru–H, 4^–^_RuSP_, and HCOO^–^ liberation, which has a low energy barrier of 4.7 kcal mol^–1^ (TS12_RuSP_ – 4^–^_RuSP,_ see Scheme S15).

In summary, the initial active intermediate 2^–^_RuSP_ can follow the *endo* or *exo* route, both of which lead to the formation of CO as the preferred kinetic product, followed by HCOO^–^ and CO. This is also true if the reaction is initiated by the tricarbonyl intermediate 2^–^_Ru_, in which case the axial CO dissociates from the hydride, also in line with the selective formation of CO. It should also be noted that a hydride intermediate is detected experimentally, which is consistent with the higher stability of the 4_RuSP_ and 4_Ru_ intermediates compared to other intermediates in the reaction.

Other alternative mechanisms were also studied for the Ru complex, including the interconversion of Ru–OCHO to Ru–COOH (Scheme S16) and the bimetallic mechanism (Scheme S17) analogous to that of the Re complex. These all involved higher free energy pathways and were thus not further considered.

## Conclusions

This work provides insights into the distinct properties of the complexes used as electrocatalysts for the reduction of CO_2_. Despite having identical ligand structures, 1_MnBr_, 1_ReCl,_ and 1_RuCl2_ exhibit diverse behaviors. During the activation process of the catalysts, 1_MnBr_ undergoes a direct two-electron reduction, while 1_ReCl_ follows a two-step reduction. This difference can be attributed to the most accessible d^7^ configuration of Mn due to its smaller crystal field splitting. 1_RuCl2_ also reacts differently, involving two cycles of reduction. The first follows a two-electron reduction, and the second a one-electron reduction, resulting in a square-planar complex with two CO ligands. In this case, the initial +2 oxidation state of Ru allows for the dissociation of a chloride anion after the second reduction, giving access to an energetically favorable d^8^ square planar configuration. The AIMD simulations conducted for Mn and Re complexes revealed that the three CO ligands rotate freely around the center, giving rise to both *endo* and *exo* conformations, depending on the position of the vacant site. While 2^–^_Re_ showed the distinct *exo* and *endo* conformers upon optimization with DFT, 2^–^_Mn_ converged to a single conformation, with a distinct location of the vacant site. Despite these differences, both Mn and Re complexes showed a preference for the *endo* conformation upon protonation of the amine and with shorter Mn···N distances compared to the nonprotonated system. The mechanistic study of the CO_2_ reduction for the three complexes showed distinct reactivity patterns. The Mn complex displays lower kinetic and thermodynamic energies in the *endo* pathway relative to the *exo*. Within the *endo*, it showed a selectivity toward the formation of HCOO^–^ over H_2_ and CO. For the Re complex, the *exo* pathway was favored, with a lower energy barrier for the formation of Re-COOH supporting CO formation through a high overpotential pathway. The Re complex can also initiate a bimetallic mechanism releasing CO due to the formation of a singly reduced species during activation. In the case of Ru, the initial active intermediate can follow the *endo* or *exo* pathway, both leading to the kinetically preferred CO formation, followed by HCOO^–^ and H_2_. This was also true if the reaction was initiated by the tricarbonyl intermediate, in which case the axial CO dissociated from the hydride intermediate.

In summary, this work showed that the nature of the metal center has a strong influence on the catalytic mechanisms of CO_2_ electroreduction. We have observed fundamental differences between the reaction pathways promoted by each metal, which, in turn, have a dramatic impact on the selectivity of the reaction. In this regard, the energies and geometries hereby reported will be highly valuable in the design of catalysts yielding higher selectivity.

## Data Availability

The data underlying this study are available in the published article and its Supporting Information.

## Supporting Information Available

Additional computational details, Figures and Tables containing natural charges, spin densities, and AIMD simulations, and energy profiles (PDF)
Geometries for all reaction intermediates and tran-sition states (XYZ)

## Supplementary Material

gg4c00046_si_001.pdf

gg4c00046_si_002.xyz
