# Supplementary material for: Metal-Dependent Mechanism of the Electrocatalytic Reduction of CO2 by Bipyridine Complexes Bearing Pendant Amines: A DFT Study
Source: ACS Org Inorg Au. 2024 Oct 11;5(1):26–36. doi: 10.1021/acsorginorgau.4c00046 (PMC11803466; doi:10.1021/acsorginorgau.4c00046)
Supplement: Supplementary file 1 — gg4c00046_si_001.pdf [file gg4c00046_si_001.pdf]

Supporting Information

Metal-Dependent Mechanism of the Electrocatalytic Reduction of CO_2 by Bipyridine Complexes Bearing Pendant Amines: A DFT Study

Mahika Luthra,[a] Abril C. Castro,[a,*] David Balcells,[a] Kim Daasbjerg[c] and Ainara Nova[a, b,*]

[a]Hylleraas Centre for Quantum Molecular Sciences, Department of Chemistry, University of Oslo, 0315, Oslo, Norway
[b]Center for Materials Science and Nanotechnology, Department of Chemistry, University of Oslo, 0315, Oslo, Norway

[c]Novo Nordisk Foundation (NNF) CO_2 Research Center, Interdisciplinary Nanoscience Center, Department of Chemistry, Aarhus University, Gustav Wieds Vej 10C, 8000 Aarhus C, Denmark

*Correspondence to a.n.flores@kjemi.uio.no (A.N.) and abril.castro@kjemi.uio.no (A.C.C.)

Table of Contents

1. Computational methods and details 2
   1.1 Workflow 2
   1.2 DFT/SMD calculations 3
   1.3 Computational study of reduction potentials 4
   1.4 Electronic structure analysis 4
   1.5 AIMD simulations methodology 6
   1.6 Discussion on the high and low-overpotential pathways 8
2. Additional figures and tables for the natural charge and spin density calculations 9
3. Additional figures and tables for AIMD calculations. 12
4. Schemes S2-S10: DFT calculations for the energy profile diagrams 14

1. Computational methods and details

1.1 Workflow

The computational methodology applied in this work is based on Density Functional Theory (DFT). The study employs classical DFT with the solvation model based on density (DFT/SMD) and ab-initio Molecular Dynamics (AIMD). Scheme S1 illustrates how these two methods have been combined for studying complexes $\mathbf{1_M}$, $\mathbf{2^-_M}$, and $\mathbf{3_M}$ (M = Mn, Re). The analysis starts with the initial intermediate $\mathbf{1_M}$, using its geometry from the crystal structure. DFT/SMD methods are used to optimize this structure. Subsequently, the active anionic intermediate $\mathbf{2^-_M}$ is formed after the 2-electron reduction and halide (X^-) dissociation. The details of how to compute the redox potential of the reactions involving electrons are provided in Section 1.3. The $\mathbf{2^-_M}$ intermediate formed plays a crucial role in the subsequent stages of the CO_2 reduction mechanism. After optimizing this intermediate using DFT/SMD, AIMD simulations are employed to investigate its flexibility and identify stable conformers from the production trajectory. These conformers are reoptimized with DFT/SMD methods, and the lowest-energy conformer among them is selected for computing the energy profiles for CO_2 reduction.

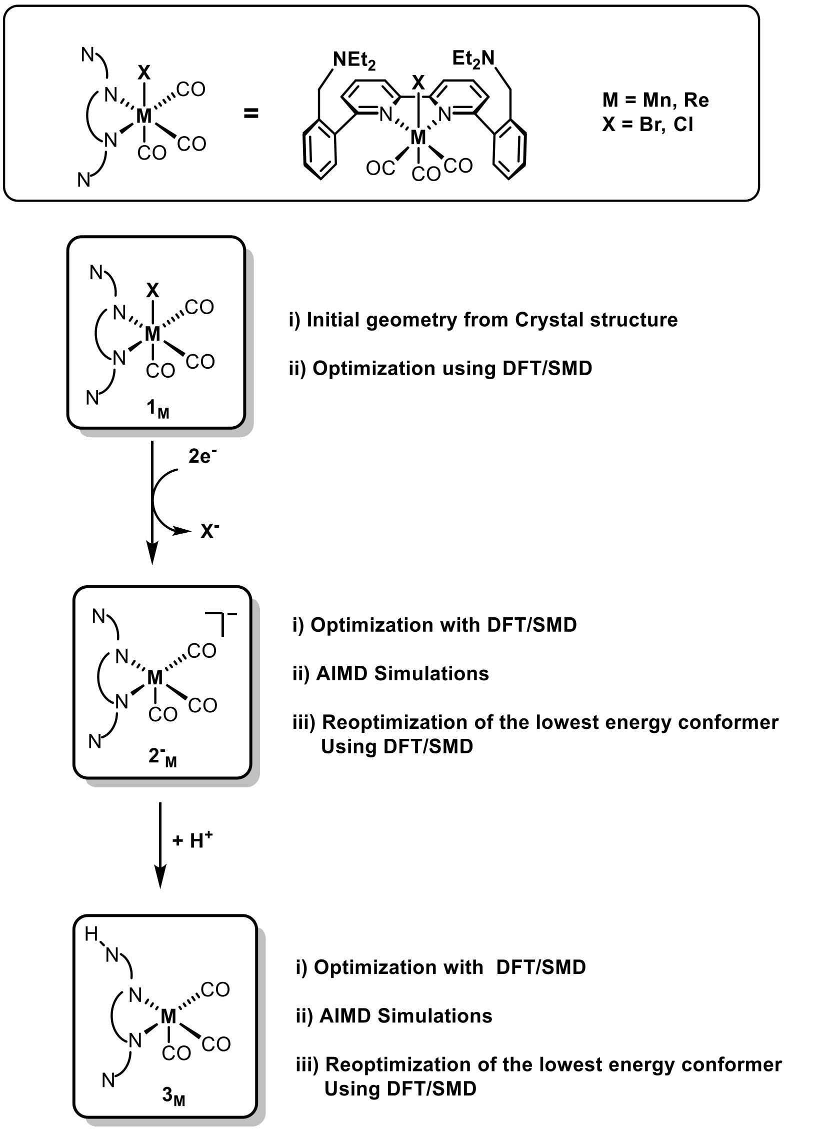

Scheme S1. Computational protocol followed for the AIMD simulations and DFT/SMD calculations.

1.2 DFT/SMD calculations

Density Functional Theory (DFT) was used in this work for the calculation of the ground energies and geometries.[1,2] The calculation of the Gibbs free energy (ΔG) was done by employing vibrational frequency calculations based on the harmonic approximation within the framework of quantum mechanics.[3]

All calculations were performed using the Gaussian 16 program, Revision C.01.[4] As shown in Table S1, a benchmark was done for the Mn-hydride complex (**4**$_{\mathbf{Mn}}$) to find the most suitable method which resembles the experimental redox potential for the reduction of the Mn-hydride intermediate. The selected functionals were B3LYP, TPSS, and TPSSh. B3LYP,[5] a widely used hybrid functional, is known for its balanced treatment of correlation and exchange, making it suitable for diverse molecular systems, including Mn-based complexes. TPSS,[6] a meta-generalized gradient approximation (meta-GGA) functional, was included for its improved treatment of non-local correlation effects, especially valuable in transition metal complexes. TPSSh,[6,7] an extension of TPSS with a 25% exact exchange, was selected to incorporate a higher fraction of Hartree-Fock exchange, enhancing its performance for systems with significant electron correlation. For the basis set, two types of basis set combinations were considered (Table S1):

i) Combination I: For the optimization of the complex, the def2-SVP (split valence polarization)[8] basis set was employed. In the case of the manganese (Mn) atom, the def2-TZVP[9] (valence triple-zeta polarization) basis set was used for the single-point calculation. For the remaining atoms, a combination of def2-SVP for optimization and def2-TZVPD[9] (valence triple-zeta polarization with diffuse functions) for the single-point calculation was used.
ii) Combination II: def2-SVP for optimization and def2-TZVP for single point calculation for all the atoms was used.

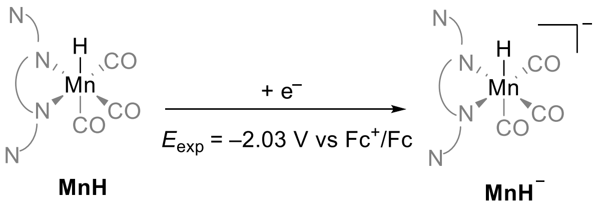

Table S1. Benchmarking for the Mn-hydride complex with different functionals and basis set combinations to find a suitable method for the DFT calculations.

Functional	Basis Set	$E°$ (V vs Fc$^+$/Fc) Experimental	$E°$ (V vs Fc$^+$/Fc) Calculated	Error (Exp-Cal)
B3LYP	I	–2.03	–1.71	–0.32
	II	–2.03	–2.32	+0.29
TPSS	I	–2.03	–1.80	–0.23
	II	–2.03	–1.81	–0.22
TPSSh	I	–2.03	–1.84	–0.19
	II	–2.03	–2.42	+0.39

The experimental redox potential of E° = -2.03 V vs Fc+/Fc for the reduction of metal hydride [MnH → MnH(-)] was used for the benchmarking. The calculation of the redox potentials was done following the procedure shown in Section 1.3. The method showing the least error in experimental and calculated values was TPSSh with the basis set combination I. Hence, this method was used for all the calculations.

All the geometries were optimized including the Grimme's D3 empirical dispersion correction.[10] Frequency calculations were performed on the optimized geometries to verify that the geometries correspond to minima or first-order saddle points (transition states) on the potential energy surface and to compute zero-point energies and thermal corrections for the reported free energies at 298 K.[11,12] The effect of the solvent was evaluated in all the calculations using the continuum Solvation Model based on Density (SMD) with the settings for acetonitrile.[13] The Gibbs free energies of all species are calculated at a standard state of 1M (sol) at 298 K.

1.3 Computational study of reduction potentials

The standard reduction potentials were calculated as follows:

For a reduction process starting from A,

$A(sol) + n\, e^{-}(g) \rightarrow A^{n-}(sol)$

where n represents the number of electrons involved and A^{n-} is the nth reduced form of A.

The reduction potential relative to the ferrocenium/ferrocene couple was calculated as

$E^\circ (A/A^{n-})\ (\text{vs } Fc^+/Fc) = E^\circ (Fc^+/Fc) - \Delta G^\circ (A/A^{n-})/nF$

where $\Delta G^\circ (A/A^{n-})$ is the change of Gibbs free energy for the reduction of A(aq), which is calculated using DFT/SMD; and F is Faraday constant (23.0605 kcal mol^{-1} V^{-1}).

1.4 Electronic structure analysis

Natural charges and spin density

In addition to the redox potential, it is important to locate the site where the incoming electron is accommodated in the complex. Essentially, this added electron can be received by either the metal center, constituting a metal-centered reduction, or it can be acquired by the ligand, leading to a ligand-centered reduction.

The methodology described above for DFT calculations was to determine the natural charges and spin densities of the various species participating in the catalyst activation process. Based on these calculations, the location of the electron was established, which, in turn, determined the number of d-electrons present on the metal center. This information served as a critical factor in the comparative assessment of the redox activity exhibited by the different metal catalysts.

The intermediates participating in the activation of the catalyst were divided into the following fragments for this analysis (Hydrogen atoms are omitted from the figures for clarity):

a) Metal Center (Mn, Ru, Re)

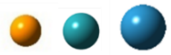

b) Halide (Br^- or Cl^-)

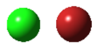

c) Bpy ligand

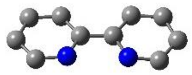

d) Three CO ligands

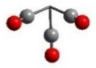

e) Other Fragments

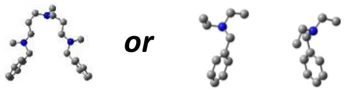

The natural charges and spin densities of these fragments are determined for the intermediates taking part in the activation of the catalysts to form the active anionic intermediate.

Spin states

The transition metal-containing complexes under investigation may manifest numerous accessible spin states or multiplicity due to the arrangement of electronic spins within the molecular orbitals. Multiplicity is defined as 2S+1, where S is the total electronic spin. These states span configurations such as singlet, doublet, triplet, or higher multiplicities depending on the number of unpaired electrons being 0, 1, 2 or higher, respectively. For instance, using crystal field theory, in an octahedral geometry for a d^6 system, the arrangement of electrons into non-bonding orbitals, $(xy)^2(xz)^2(yz)^2$, often leads to a singlet or low spin state (Figure 2.1). However, if the energy of anti-bonding orbitals (x^2-y^2, z^2) is sufficiently reduced to surpass the pairing energy due to enhanced exchange interactions, a triplet spin state $(xy)^2(xz)^2(yz)^1$ $(z^2)^1(x^2\text{-}y^2)^0$, might exhibit lower energy (see Figure 2.1). Higher spin states involve more parallel electron spins, intensifying exchange interactions.[14] The favorability of high or low-spin states depends on the magnitude of the separation between the d-orbitals splitting (Δ_o), which in turn depends on the ligand field strength generated by the surrounding ligands. Predicting the most favorable electron distribution and, consequently, the preferred spin states pose a challenge, complicating accurate treatment using computational methods such as DFT.

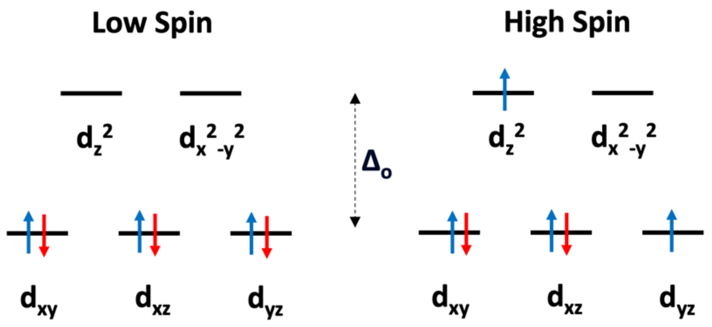

Figure S1. Schematic representation of d-orbital energy levels for a low spin (left) and a high-spin (right) transition-metal complex.

The first-row transition metals, in general, show a larger sensitivity to how the electrons are distributed over the d-orbitals than the corresponding transition metals in higher rows of the periodic table, due to their smaller atomic sizes and effective nuclear charges, which results in stronger interactions between the ligands and d-orbitals, leading to more pronounced variations in energy levels. Moreover, their orbitals are less diffuse, resulting in higher pairing energy compared to second and third-row metals, which leads to a broader range of accessible spin states in first-row transition metal complexes.[15]

In the context of this study involving complexes featuring Mn, Re, and Ru as metal centers, the energies associated with various spin states for the intermediates $\mathbf{1_{Mn}}$, $\mathbf{2_{Mn}}$, and $\mathbf{2^{-}_{Mn}}$ within the Mn complex were computed using a spin ladder approach, as detailed in Table S2.

Table S2. Spin ladder for **1_{Mn}**, **2_{Mn}** and **2^-_{Mn}**; all energies are in kcal mol^{-1}. SM refers to spin multiplicity.

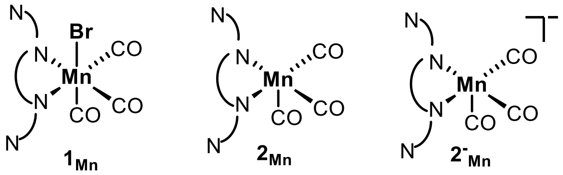

**1_{Mn}**	**ΔG**	**2_{Mn}**	**ΔG**	**2^-_{Mn}**	**ΔG**
**SM = 1**	0.0	**SM = 2**	0.0	**SM = 1**	0.0
**SM = 3**	35.2	**SM = 4**	27.6	**SM = 3**	14.8
**SM = 5**	43.8			**SM = 5**	43.1

The observed trend reveals that the most stable spin state across all complexes corresponds to the lowest multiplicity state. This outcome aligns with expectations, given that CO serves as a strong field ligand, which increases the separation between the d-orbitals (Δ_o), thereby favoring a preference for the low-spin state.

1.5 AIMD simulations methodology

AIMD simulations[16] of the Mn (**1_{Mn}**) and Re (**1_{Re}**) complexes on the active intermediates (**2^-_M**) and their protonated counterparts (**3_M**) (Scheme S1) were run in an explicit acetonitrile (CH_3CN) solvent according to the Born-Oppenheimer approximation using the *CP2K* program package.[16] The initial model systems were created using the *PACKMOL* package (Figure S2),[18] which consisted of the complex under study (previously optimized at the TPSSh level) surrounded by either 50 CH_3CN molecules in a cubic box of 18 Å (for **2^-_{Mn}**) or by 25 CH_3CN molecules in a cubic box of 15.5 Å (for **2^-_{Re}**), to reproduce the appropriate density of 0.786 g mL^{-1}. The number of solvent molecules and edge length of the cubic box for both varies because of the difference in size and molar masses of **2^-_{Mn}** (617.23 g mol^{-1}) and **2^-_{Re}** (749.25 g mol^{-1}). The simulation cell was treated under periodic boundary conditions.

The electronic problem is solved at the DFT level of theory, with the PBE exchange-correlation functional.[19] All atoms are described with a DZVP basis set, and with GTH pseudopotentials. Auxiliary plane-waves basis set is cut-off to 200 Ry. [17] Dispersion forces were considered using Grimme's D3 model.[18] The initial structure was relaxed using a microcanonical (NVE) ensemble, until an average temperature of 298 K was reached. After equilibration, the simulation was run using a canonical (NVT) ensemble with a temperature of 298 K maintained with a CSVR algorithm.[19] Core electrons were described using pseudopotentials of the Goedecker-Teter-Hutter type.[20] The trajectories were extended up to 25 ps with a time step of 0.25 fs. The production trajectories with an NVT ensemble were used to extract the lowest energy conformers, as shown in Figure S3 and S4. These conformers were then optimized with DFT/SMD calculations using TPSSh functional and def2SVP/def2TZVP basis set. Additionally, DFT/SMD calculations were also performed

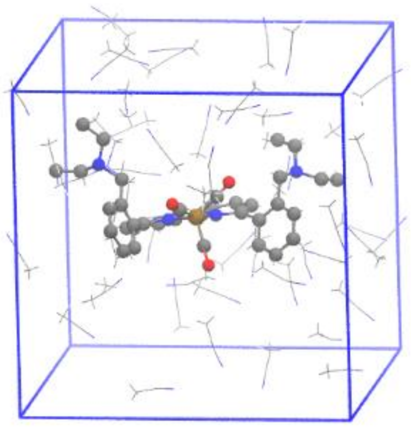

Figure S2. Representation of the complex ($\mathbf{2^{-}_{Mn}}$) in a box with explicit CH_3CN solvent.

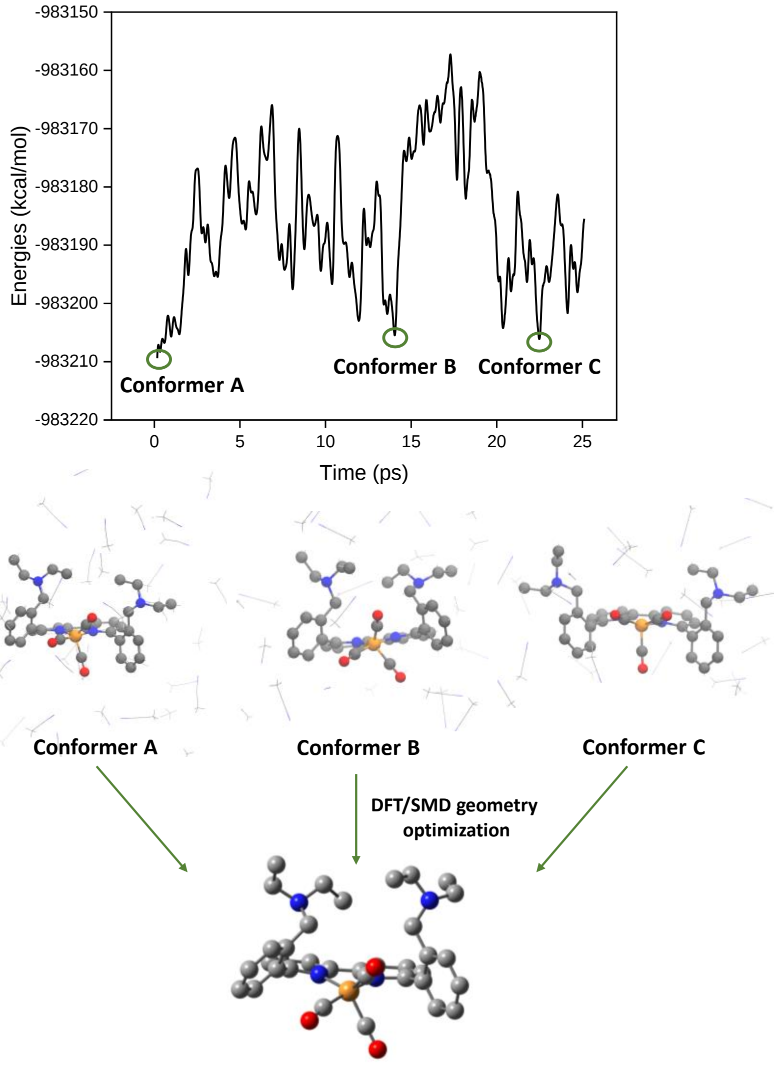

Figure S3. Methodology to extract lowest energy conformers from the AIMD trajectory of Mn complex.

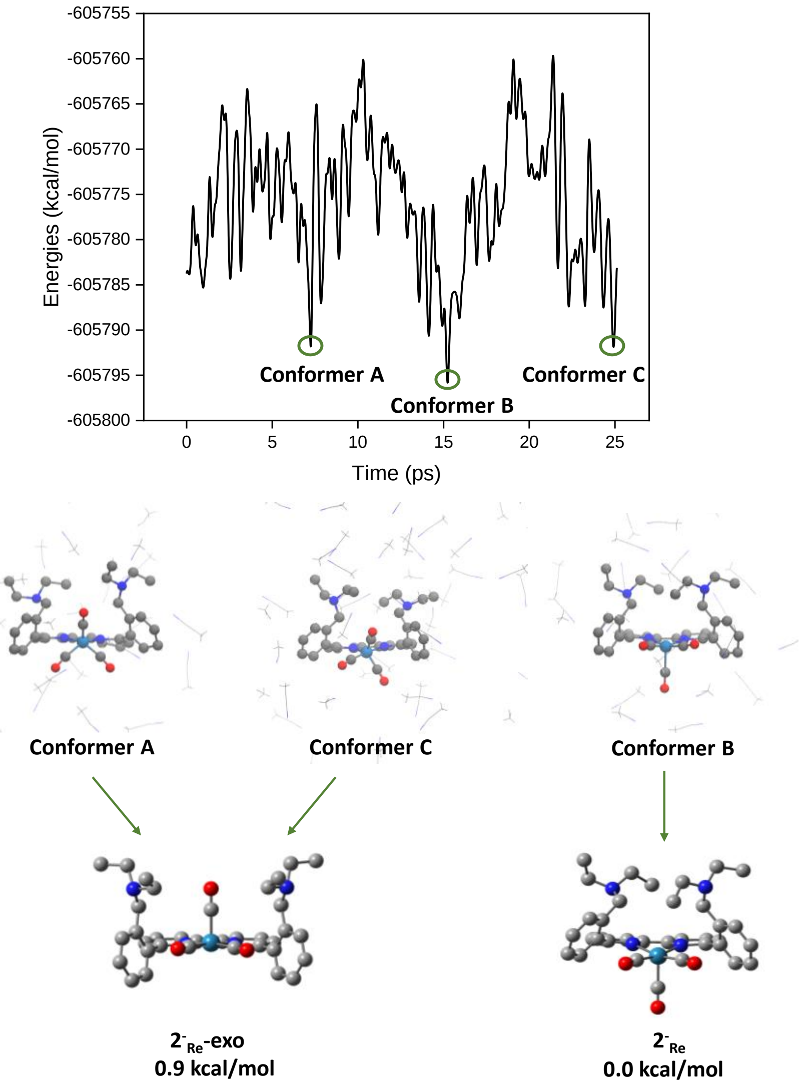

Figure S4. Methodology to extract lowest energy conformers from the AIMD trajectory of Re complex

1.6 Discussion on the high and low-overpotential pathways

In Scheme 5 and 7, the term "low overpotential pathway" is used to refer to the path that only requires the reduction of the initial complex (e. g. $\mathbf{2^{-}_{Re}}$, in Scheme 5). In contrast, the "high overpotential pathway" requires the reduction of one of the reaction intermediates from the "low overpotential pathway" (e. g. $\mathbf{9^{-}_{Re}}$**-exo**, in Scheme 5). It is assumed that exergonic reactions involving low energy barriers will proceed without the further reduction of the involved species. Instead, reactions with highly stable intermediates, which can accumulate, can be further reduced if the applied potential is higher than their redox potential. In Scheme 9, the redox potential of $\mathbf{9^{-}_{Re}}$**-exo** is -1.7 V, while the applied potential is −2.37 V.[21]

2. Additional figures and tables for the natural charge and spin density calculations

Table S3. Natural charge and spin density analysis for the intermediates involved in the redox activation process of the Mn complex.

Fragment	Natural Charge (*e*)				Spin Density (e/Å^3)			
	$\mathbf{1_{Mn}}$	$\mathbf{1^-_{Mn}}$	$\mathbf{2_{Mn}}$	$\mathbf{2^-_{Mn}}$	$\mathbf{1_{Mn}}$	$\mathbf{1^-_{Mn}}$	$\mathbf{2_{Mn}}$	$\mathbf{2^-_{Mn}}$
**Mn**	-0.53	-0.52	-1.35	-1.62	0	0.006	0.7	0
**Bromide**	-0.53	-0.57	-	-	0	0.02	-	-
**Bipyridine**	0.37	-0.43	0.43	-0.13	0	0.97	0.14	0
**3 CO**	0.62	0.54	0.86	0.78	0	0.01	0.14	0
**Other**	0.07	0.02	0.51	0.40	0	0	0.02	0

Table S4. Natural charge and spin density analysis for the intermediates involved in the redox activation process of the Re complex.

Fragment	Natural Charge (*e*)				Spin Density (e/Å^3)			
	$\mathbf{1_{Re}}$	$\mathbf{1^-_{Re}}$	$\mathbf{2_{Re}}$	$\mathbf{2^-_{Re}}$	$\mathbf{1_{Re}}$	$\mathbf{1^-_{Re}}$	$\mathbf{2_{Re}}$	$\mathbf{2^-_{Re}}$
**Re**	-0.03	0.002	0.25	-0.56	0	-0.01	0.04	0
**Chloride**	-0.60	-0.62	-	-	0	0.01	-	-
**Bipyridine**	0.31	-0.49	-0.39	-0.67	0	0.99	0.82	0
**3 CO**	0.23	0.12	0.08	0.78	0	0.03	0.09	0
**Other**	0.09	-0.03	0.51	0.40	0	-0.03	0.02	0

Table S5. Natural charge and spin density analysis for the intermediates involved in the redox activation process of the Ru complex for the release of the first chloride.

Fragment	Natural Charge (*e*)				Spin Density (e/Å^3)			
	$\mathbf{1}_{Ru}$	$\mathbf{1}^{-}_{Ru}$	$\mathbf{2}_{RuCl}$	$\mathbf{2}^{-}_{RuCl}$	$\mathbf{1}_{Ru}$	$\mathbf{1}^{-}_{Ru}$	$\mathbf{2}_{RuCl}$	$\mathbf{2}^{-}_{RuCl}$
**Ru**	-0.75	-0.75	-0.48	-0.79	0	-0.01	0.76	0
**Chloride**	-0.74	-0.78	-0.54	-0.56	0	0.01	0.12	0
**Bipyridine**	0.59	-0.19	0.42	0.04	0	0.95	0.06	0
**2 CO**	0.77	0.70	0.45	0.27	0	0.04	0.02	0
**Other**	0.13	0.02	0.15	0.04	0	0.01	0.04	0

Table S6. Natural charge and spin density analysis for the intermediates involved in the redox activation process of the Ru complex for the release of the second chloride.

Fragment	Natural Charge (*e*)			Spin Density (e/Å^3)		
	$\mathbf{2}^{-}_{RuCl}$	$\mathbf{2}_{RuSP}$	$\mathbf{2}^{-}_{RuSP}$	$\mathbf{2}^{-}_{RuCl}$	$\mathbf{2}_{RuSP}$	$\mathbf{2}^{-}_{RuSP}$
**Ru**	-0.79	-0.62	-0.77	0	0	0.01
**Chloride**	-0.56	-	-	0	-	-
**Bipyridine**	0.04	0.28	-0.40	0	0	0.90
**2 CO**	0.27	0.22	0.15	0	0	0.07
**Other**	0.04	0.12	0.02	0	0	0.02

Table S7. Comparison of TPSSh, B3LYP and PBE0 functionals for natural charge and spin densities of **2_{Re}** and **2^-_{Re}**

Fragment	Natural Charge (*e*)						Spin Density (e/Å^3)					
	2_{Re}			2^-_{Re}			2_{Re}			2^-_{Re}		
	TPSSh	B3LYP	PBE0	TPSSh	B3LYP	PBE0	TPSSh	B3LYP	PBE0	TPSSh	B3LYP	PBE0
Re	0.25	-0.28	-0.33	-0.56	-0.52	-0.59	0.04	0.06	0.06	0	0	0
Bipyridine	-0.39	-0.25	-0.31	-0.67	-0.71	-1.35	0.82	0.84	0.85	0	0	0
3 CO	0.08	0.44	0.49	0.78	0.26	0.32	0.09	0.07	0.06	0	0	0
Other	0.51	0.09	0.15	0.40	-0.03	0.62	0.02	0.03	0.03	0	0	0

Table S8. Natural charge and spin density analysis for the intermediates involved in the reaction mechanism of Re complex after the formation of **2^-_{Re}**.

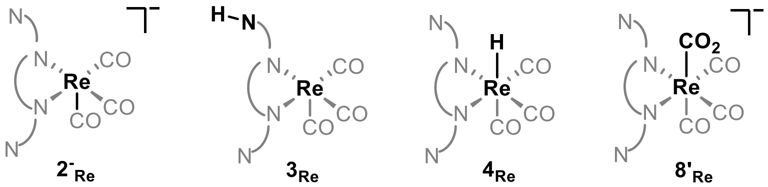

Fragment	Natural Charge (*e*)				Spin Density (e/Å^3)			
	2^-_{Re}	3_{Re}	4_{Re}	$8'_{Re}$	2^-_{Re}	3_{Re}	4_{Re}	$8'_{Re}$
Re	-0.56	-0.63	-1.01	-0.94	0	0	0	0
Hydride/CO_2	-	0.49	-0.12	-0.69	-	0	0	0
Bipyridine	-0.67	-0.55	0.45	0.16	0	0	0	0

**3 CO**	0.78	0.23	0.56	0.42	0	0	0	0
**Other**	0.40	0.46	0.12	0.05	0	0	0	0

3. Additional figures and tables for AIMD calculations.

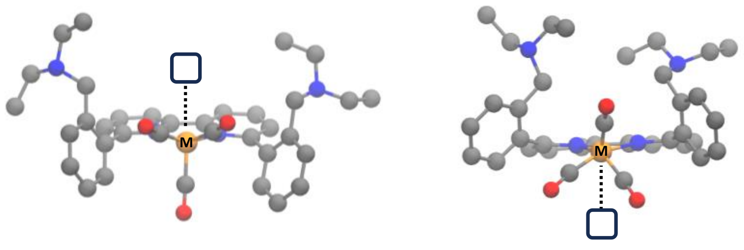

endo ***exo***

Figure S5. Representation of the *endo* and *exo* isomers obtained during the AIMD simulation of the $\mathbf{2^{-}_{M}}$ intermediate for Mn and Re complexes.

Table S9. Analysis of the M···N(left) and M···N(right) bond distances (Å) for the $\mathbf{2^{-}_{M}}$ intermediate through AIMD simulations (M = Mn, Re).

	$\mathbf{2^{-}_{Mn}}$ **Mn···N (Å)**		$\mathbf{2^{-}_{Re}}$ **Re···N (Å)**	
	**N(left)**	**N(right)**	**N(left)**	**N(right)**
**Minimum**	4.47	4.72	4.29	4.26
**Maximum**	6.95	7.30	6.23	6.27
**Amplitude**	2.48	2.58	1.94	2.01
**Average**	5.81	5.80	5.31	5.30

Table S10. Analysis of the M···N(left), M···N(right), and M···H(left) bond distances (Å) for the $\mathbf{3_{M}}$ intermediate through AIMD simulations (M = Mn, Re).

	$\mathbf{3_{Mn}}$ **Mn···N/H**			$\mathbf{3_{Re}}$ **Re···N/H**		
	**N(left)**	**H(left)**	**N(right)**	**N(left)**	**H(left)**	**N(right)**
**Minimum**	3.19	2.08	4.36	3.26	2.17	5.08

**Maximum**	4.40	3.40	6.78	4.41	3.42	6.90
**Amplitude**	1.21	1.33	2.42	1.15	1.25	1.82
**Average**	3.60	2.56	5.82	3.75	2.70	5.89

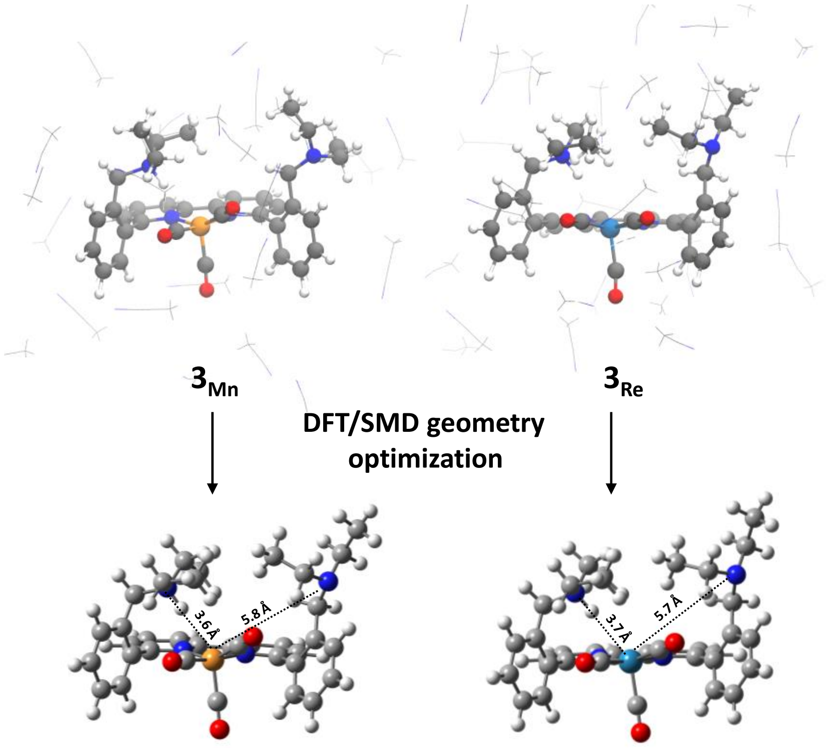

Figure S6. Isolation of lowest energy conformers of $\mathbf{3_M}$ (M = Mn, Re) found by AIMD simulations and after a DFT/SMD geometry optimization.

4. Schemes S2-S19: DFT calculations for the energy profile diagrams

Scheme S2. Gibbs energy profile (kcal mol^{-1}) for the reduction of CO_2 with TFE as a proton source to three different products: H_2 (green), $HCOO^-$ (red), and CO (blue) using the Mn complex from the *endo* isomer. All energies are considered at a redox potential of -1.55 V vs Fc^+/Fc.

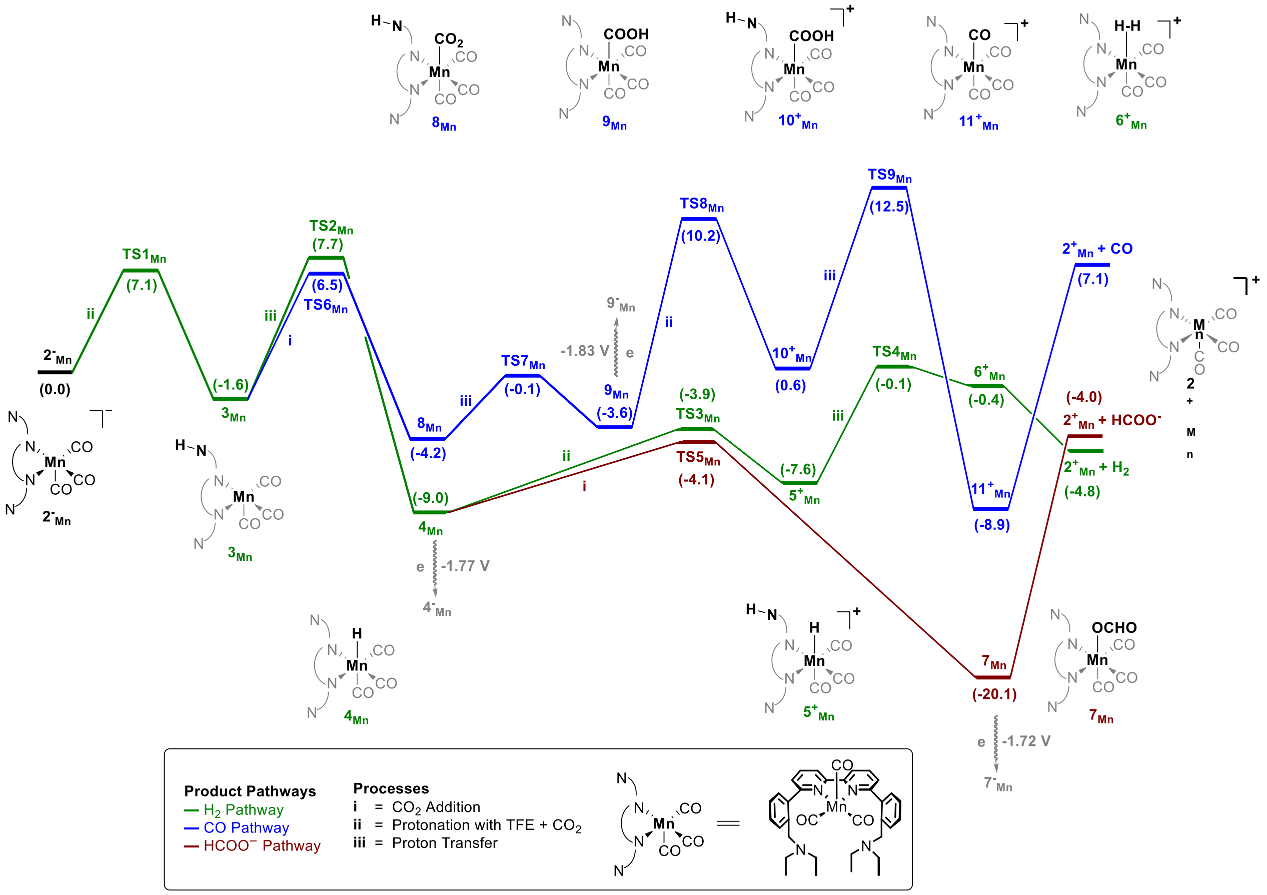

Scheme S3. Gibbs energy profile (kcal mol^{-1}) for the reduction of CO_2 with TFE as a proton source to H_2 (green) and $HCOO^-$ (red) starting with the intermediate $\mathbf{4^-_{Mn}}$. All energies are considered at a redox potential of -1.77 V vs Fc^+/Fc.

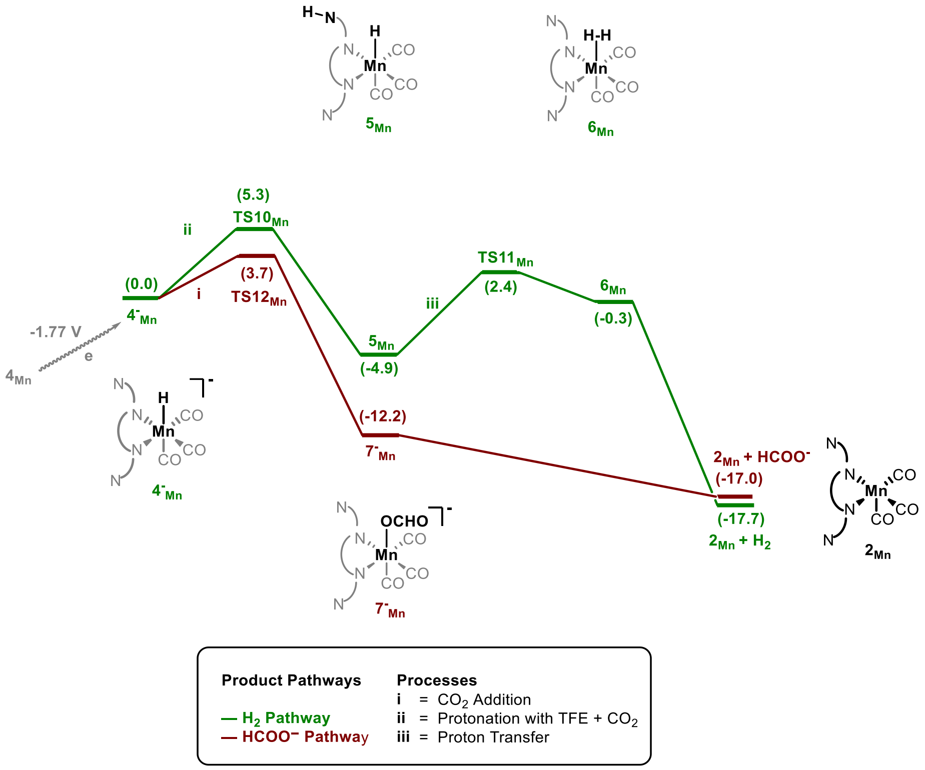

Scheme S4. Gibbs energy profile (kcal mol^{-1}) for the reduction of CO_2 with TFE as a proton source to CO (blue) starting with the intermediate $\mathbf{9^-_{Mn}}$. All energies are considered at a redox potential of -1.83 V vs Fc^+/Fc.

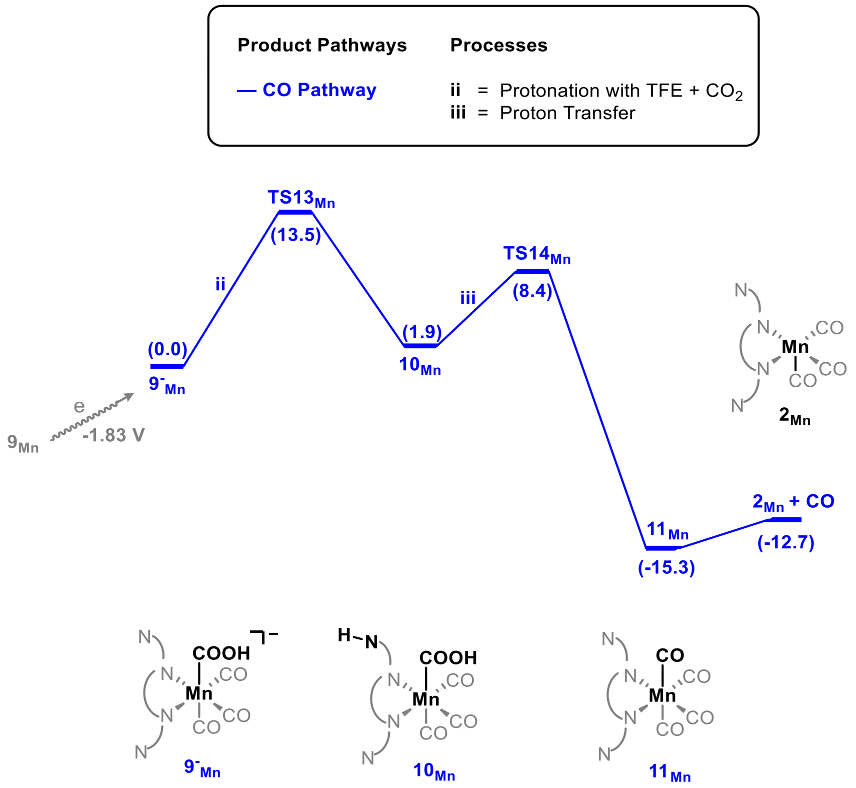

Scheme S5. Gibbs energy profile (kcal mol[-1]) for the reduction of CO_2 with TFE as a proton source to three different products: H_2 (green), $HCOO^-$ (red), and CO (blue) using the Mn complex from the *exo* isomer. All energies are considered at a redox potential of -1.55 V vs Fc^+/Fc.

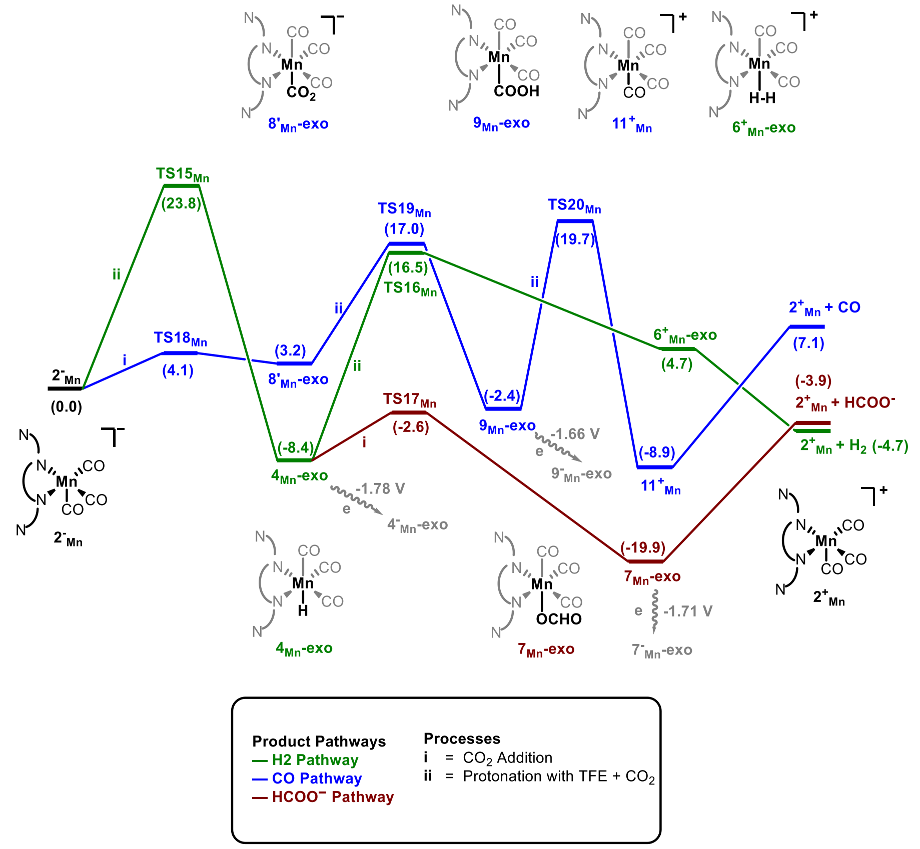

Scheme S6. Gibbs energy profile (kcal mol[-1]) for the reduction of CO_2 with TFE as a proton source to CO (blue) starting with the intermediate **9^-_{Mn}-exo**. All energies are considered at a redox potential of -1.66 V vs Fc^+/Fc.

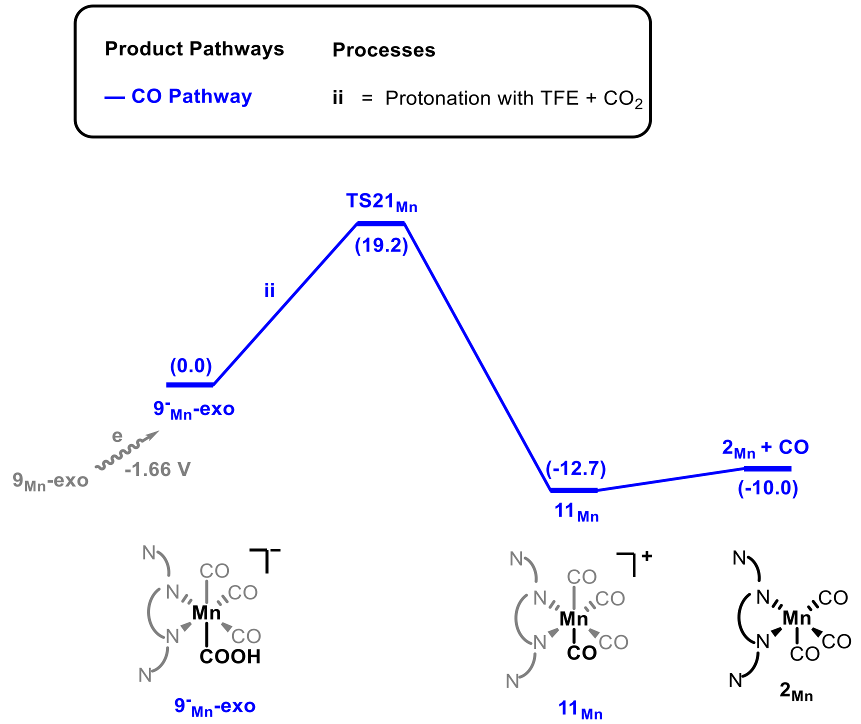

Scheme S7. Gibbs energy profile (kcal mol^{-1}) for the reduction of CO_2 with TFE as a proton source to three different products: H_2 (green), $HCOO^-$ (red), and CO (blue) using the Re complex from the *endo* isomer. All energies are considered at a redox potential of -1.70 V vs Fc^+/Fc.

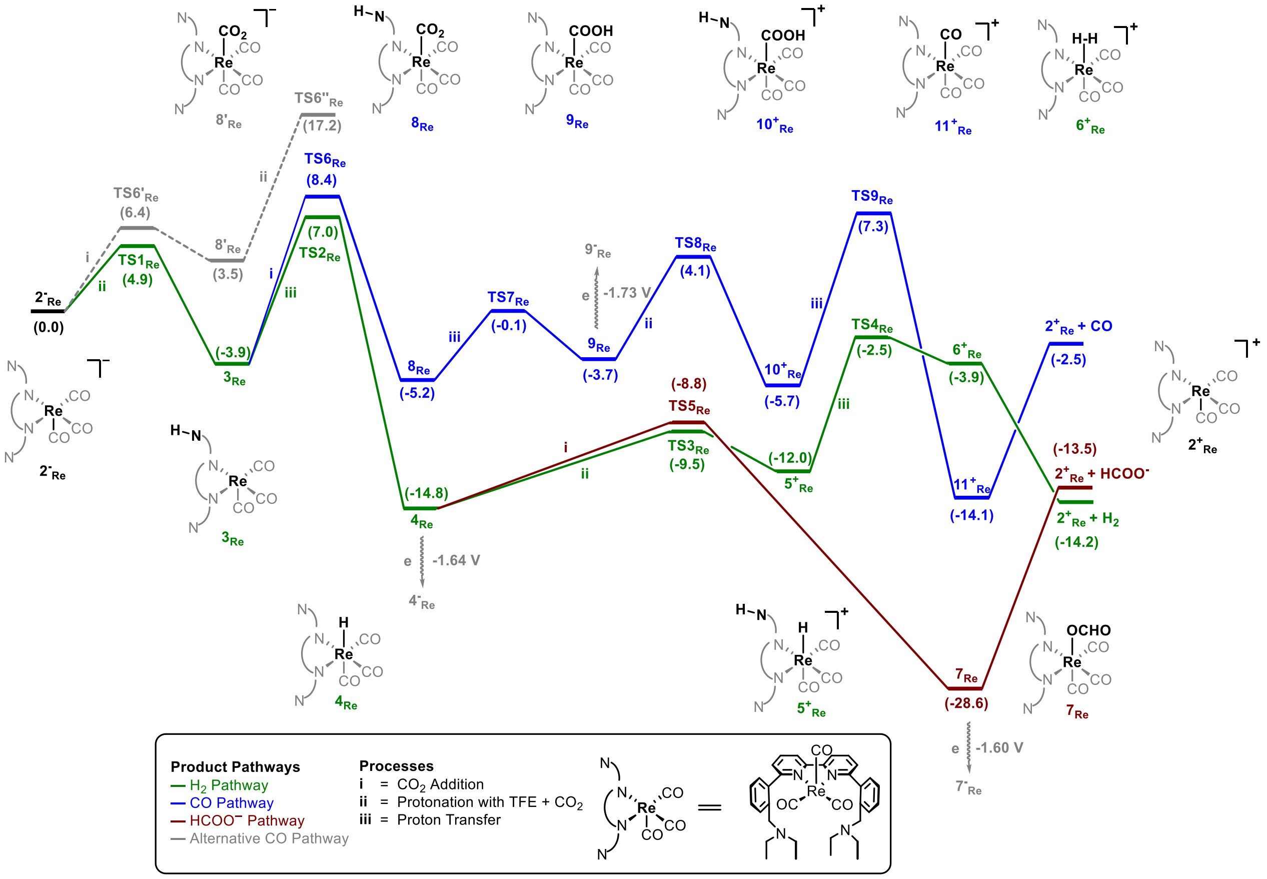

Scheme S8. Gibbs energy profile (kcal mol^{-1}) starting from **4$^-_{Re}$** to form $HCOO^-$ (Red) and H_2 (Green) for the Re complex at a redox potential of -1.64 V relative to Fc^+/Fc.

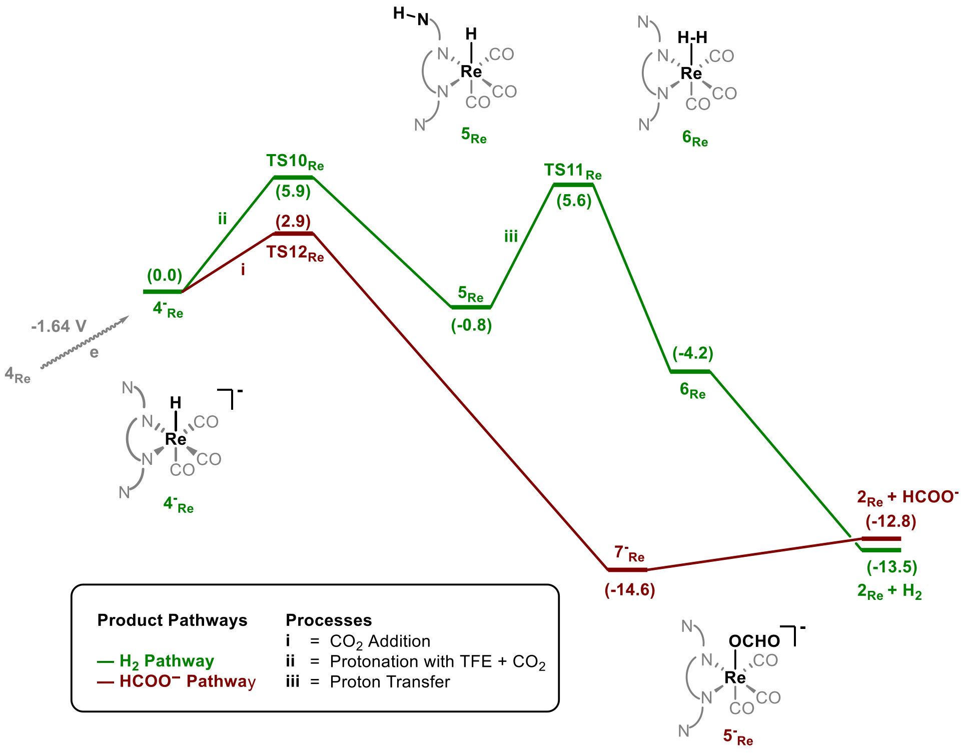

Scheme S9. Gibbs energy profile (kcal mol[-1]) starting from **9**$^-_{\mathbf{Re}}$ to form CO (blue) for the Re complex at a redox potential of -1.73 V relative to Fc$^+$/Fc.

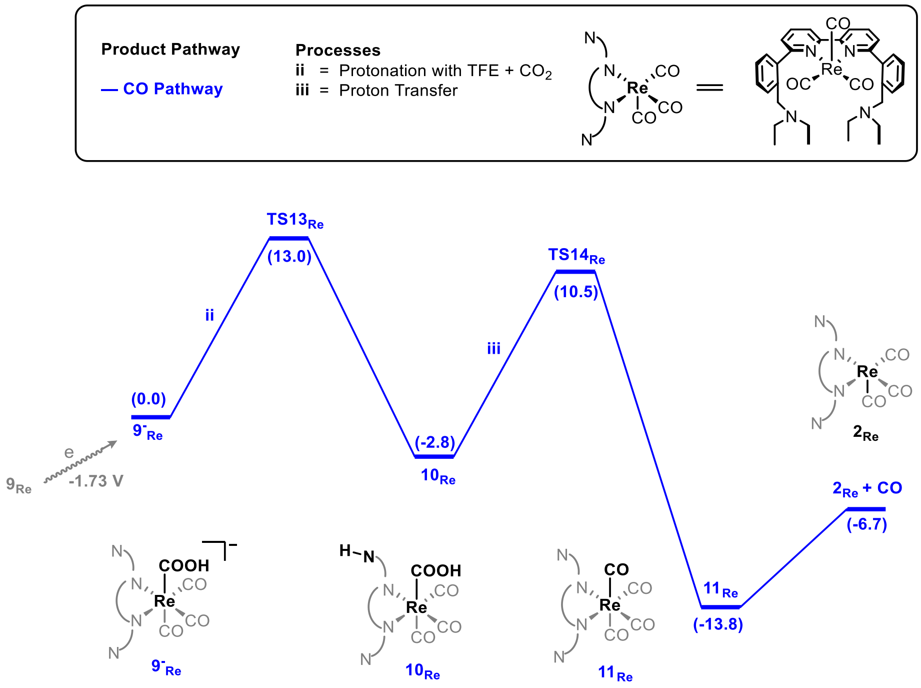

Scheme S10. Gibbs energy profile (kcal mol[-1]) calculated from the square planar complex **2**$^-_{\mathbf{RuSP}}$ from the *exo* direction. All energies are considered at a redox potential of -1.67 V vs Fc$^+$/Fc.

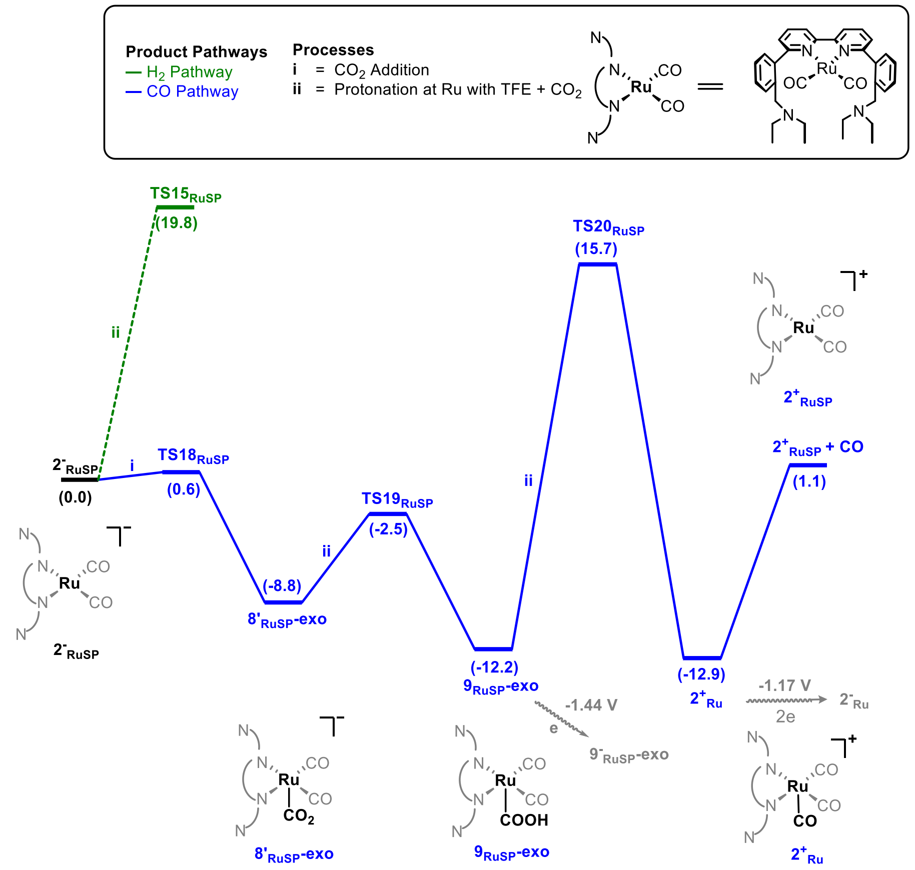

Scheme S11. Gibbs energy profile (kcal mol^{-1}) calculated starting from **9^{-}$_{RuSP}$-exo** for the formation of CO (blue). All energies are considered at a redox potential of -1.44 V vs Fc$^+$/Fc.

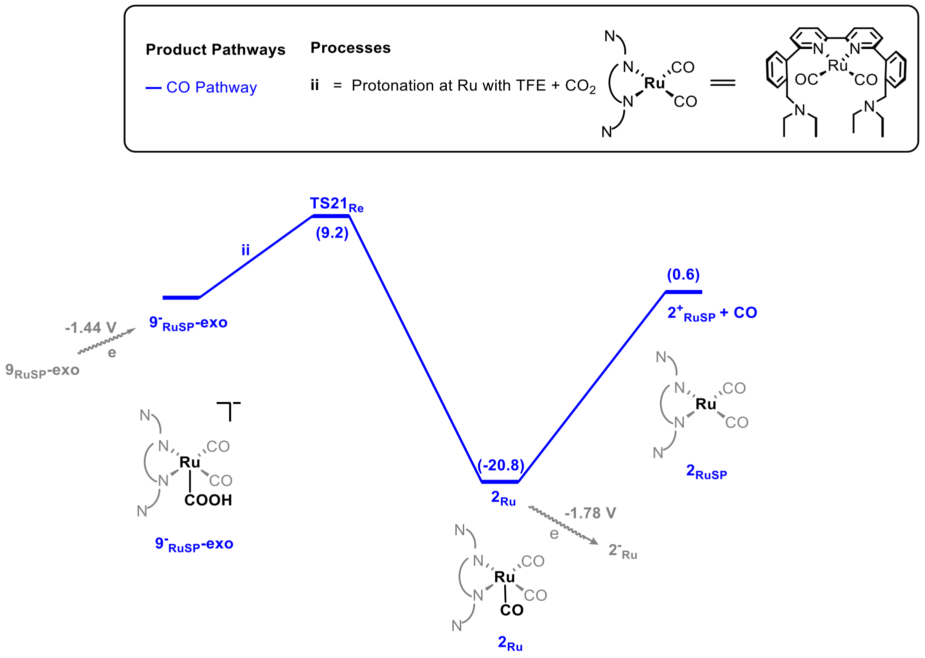

Scheme S12. Gibbs energy profile (kcal mol^{-1}) for the reduction of CO_2 with TFE as a proton source to three different products: H_2 (green), $HCOO^-$ (red), and CO (blue) using the Ru complex with three CO ligands. All energies are considered at a redox potential of -1.78 V vs Fc$^+$/Fc.

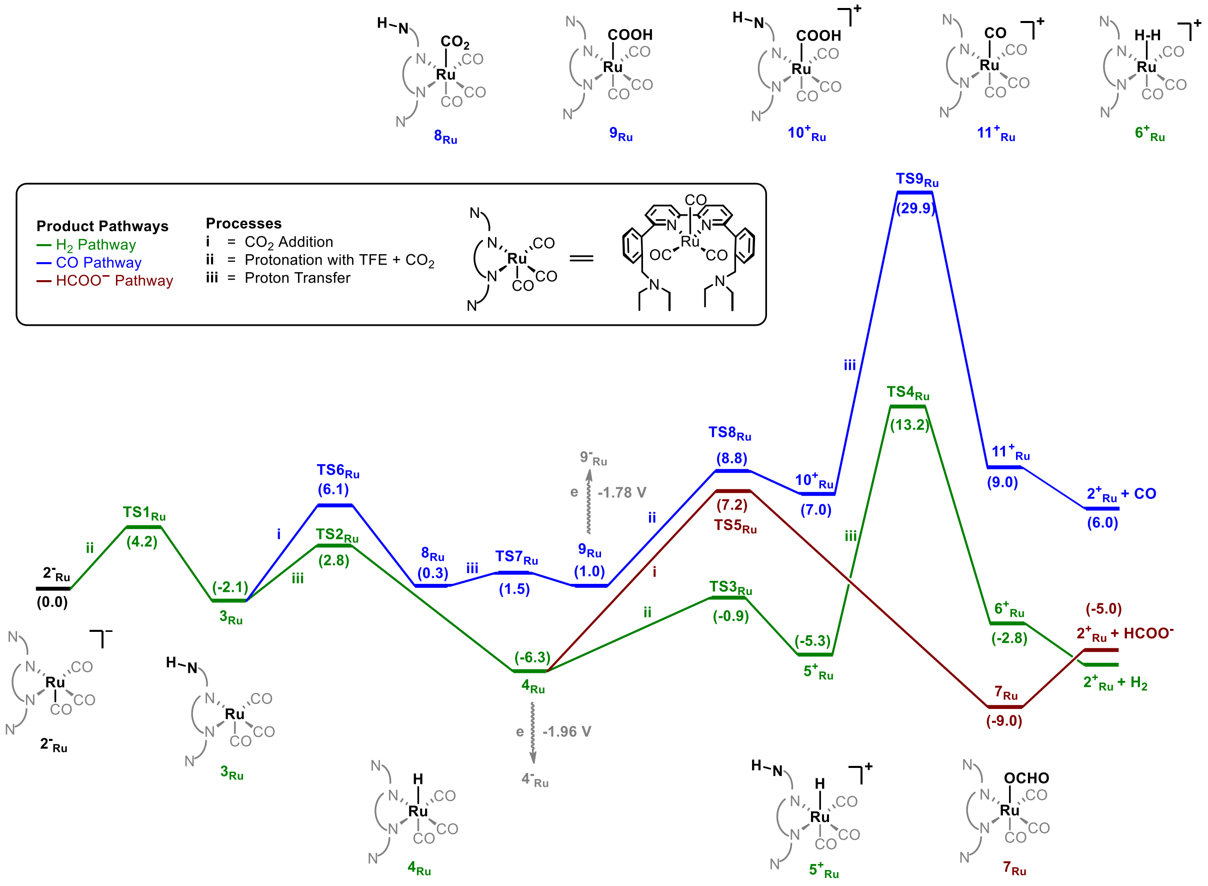

Scheme S13. Gibbs energy profile (kcal mol^{-1}) for the formation of H_2 (green) and $HCOO^-$ (red) starting from $\mathbf{4^-_{Ru}}$ at a redox potential of -1.96 V.

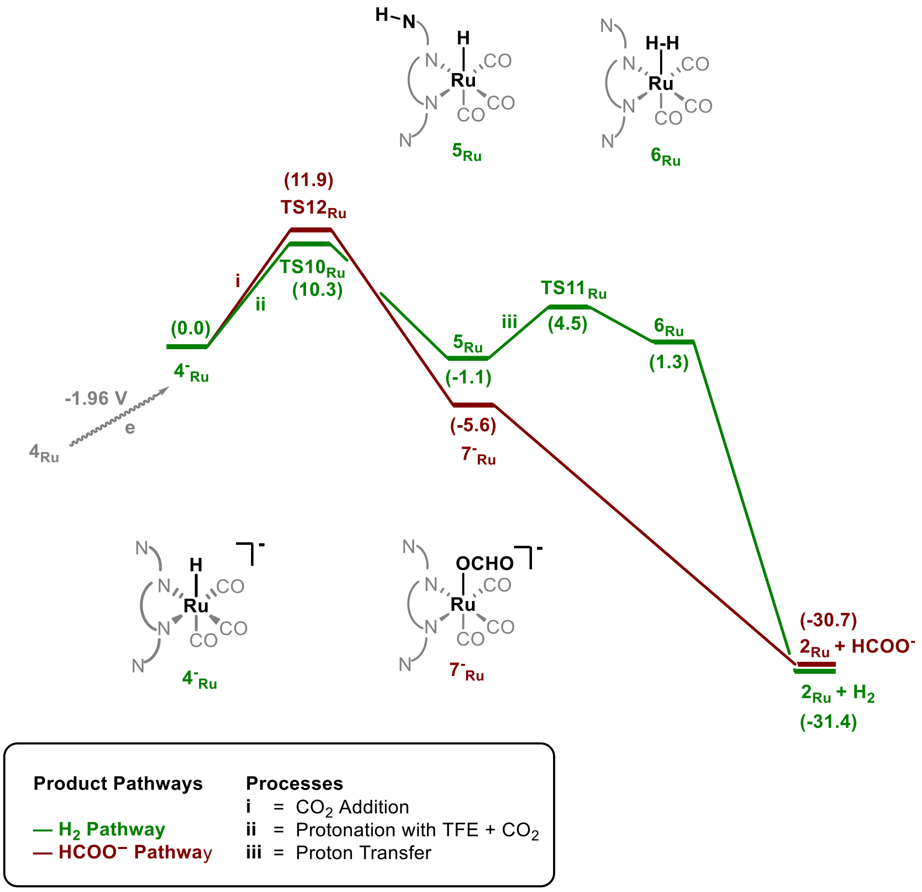

Scheme S14. Gibbs energy profile (kcal mol^{-1}) for the formation of CO (blue) starting from $\mathbf{9^{-}_{Ru}}$ at a redox potential of -1.78 V.

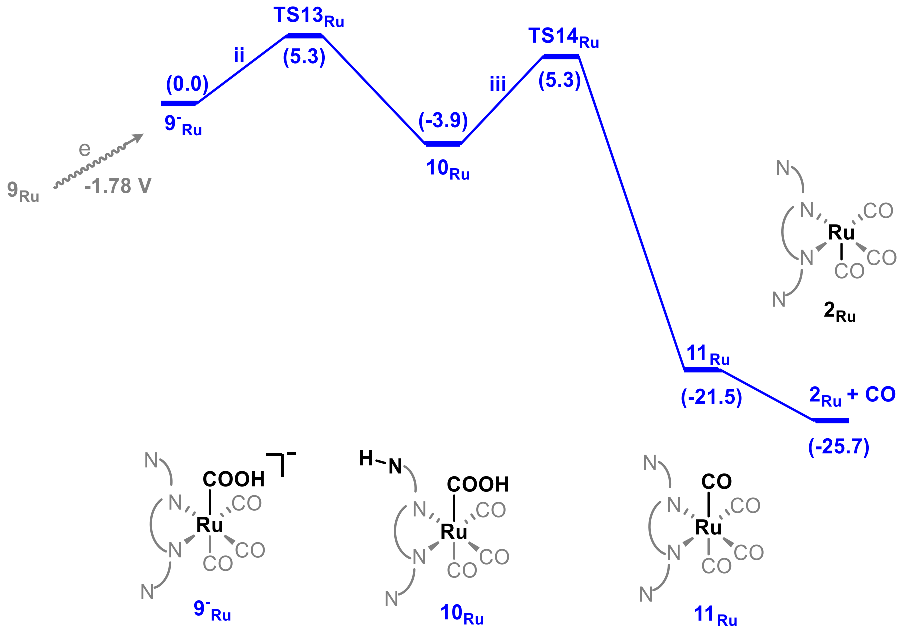

Scheme S15. Gibbs energy profile (kcal mol^{-1}) for the three product pathways: CO (blue), $HCOO^-$ (red) and H_2 (green) starting from $\mathbf{4^-_{Ru}}$. Solid lines represent the most preferable pathway, while dashed lines indicate higher energy pathways.

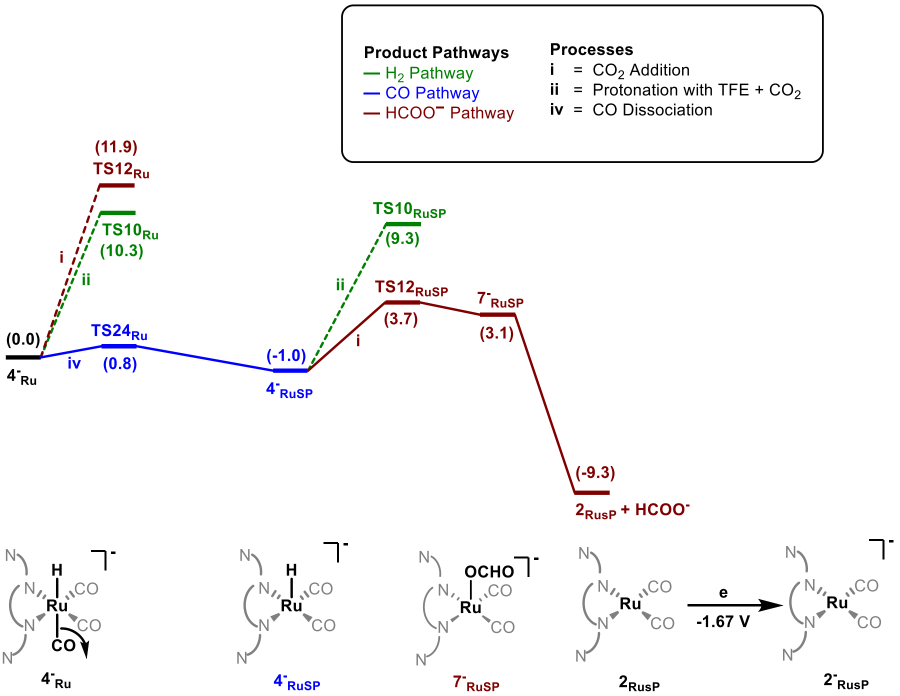

Scheme S16. Representation of the conversion of Ru-OCHO intermediate to the Ru-COOH intermediate for the formation of CO. The thermodynamic energies are shown in green, and the kinetic energy barriers are shown in red (kcal mol^{-1}).

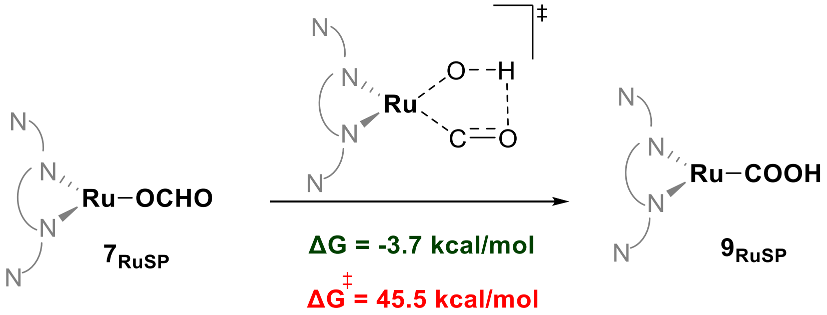

Scheme S17. Bimetallic mechanism for the Ru complex starting with $\mathbf{2^-_{RuSP}}$ intermediate. The energies in blue represent the thermodynamic free energies of the intermediates in kcal mol^{-1}.

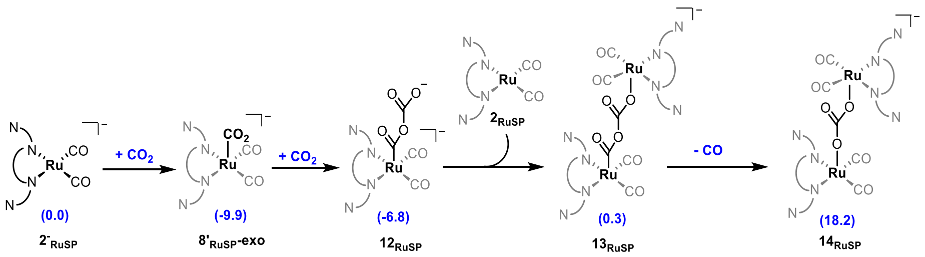

Scheme S18. Schematic representation of N-to-C proton transfer to form Mn-OCHO intermediate to release $HCOO^-$. Energies are in kcal mol^{-1} and calculated by taking $\mathbf{2}^-_{Mn}$ as 0 kcal mol^{-1}.

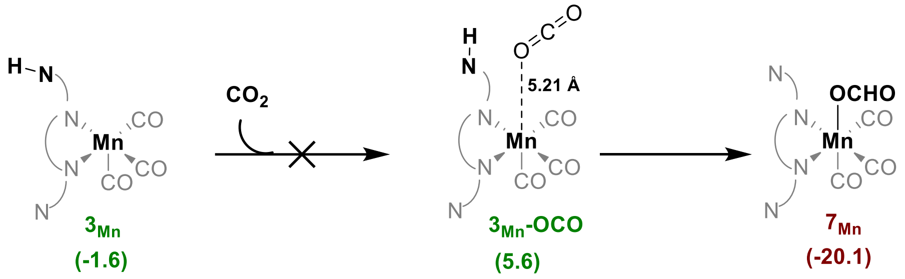

Scheme S19. Schematic representation of solvent (CH_3CN) coordination to the doubly reduced $\mathbf{2}_{Re}$ complex.

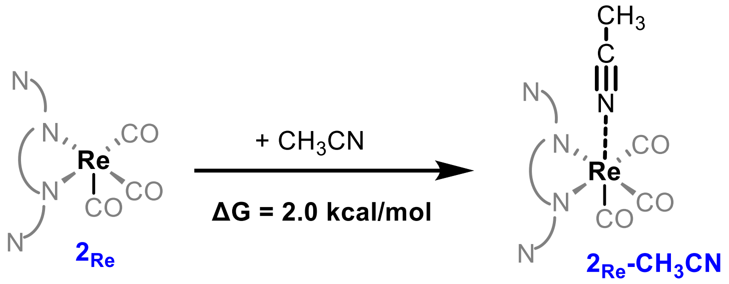

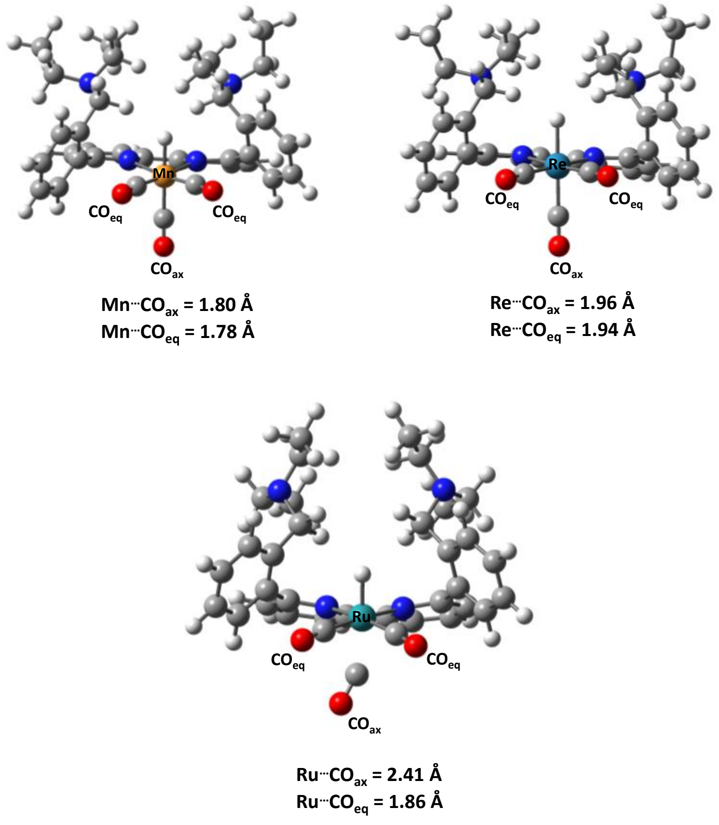

Figure S7. DFT-optimized geometries for $\mathbf{4}^-_{Mn}$, $\mathbf{4}^-_{Re}$, and $\mathbf{4}^-_{Ru}$ showing the bond distances between the metal center and the CO (axial) and CO (equatorial) ligands.

References

(1) Koch, W., Holthausen, M. C. . *A Chemist's Guide to Density Functional Theory*; Wiley, 2001.
(2) Cramer, C. J. *Essentials of computational chemistry*; Wiley, 2004.
(3) Monacelli, L.; Bianco, R.; Cherubini, M.; Calandra, M.; Errea, I.; Mauri, F. The stochastic self-consistent harmonic approximation: calculating vibrational properties of materials with full quantum and anharmonic effects. *Journal of Physics: Condensed Matter* **2021**, *33* (36), 363001. DOI: 10.1088/1361-648x/ac066b.
(4) *Gaussian 16 Rev. C.01*; Wallingford, CT, 2016. (accessed.
(5) Becke, A. D. Density-functional exchange-energy approximation with correct asymptotic behavior. *Physical Review A* **1988**, *38* (6), 3098-3100. DOI: 10.1103/PhysRevA.38.3098.
(6) Tao, J.; Perdew, J. P.; Staroverov, V. N.; Scuseria, G. E. Climbing the Density Functional Ladder: Nonempirical Meta--Generalized Gradient Approximation Designed for Molecules and Solids. *Physical Review Letters* **2003**, *91* (14), 146401. DOI: 10.1103/PhysRevLett.91.146401.
(7) Staroverov, V. N.; Scuseria, G. E.; Tao, J.; Perdew, J. P. Comparative assessment of a new nonempirical density functional: Molecules and hydrogen-bonded complexes. *The Journal of Chemical Physics* **2003**, *119* (23), 12129-12137. DOI: 10.1063/1.1626543.
(8) Eichkorn, K.; Treutler, O.; Öhm, H.; Häser, M.; Ahlrichs, R. Auxiliary basis sets to approximate Coulomb potentials. *Chemical Physics Letters* **1995**, *240* (4), 283-290. DOI: 10.1016/0009-2614(95)00621-a.
(9) Weigend, F.; Ahlrichs, R. Balanced basis sets of split valence, triple zeta valence and quadruple zeta valence quality for H to Rn: Design and assessment of accuracy. *Physical Chemistry Chemical Physics* **2005**, *7* (18), 3297-3305, 10.1039/B508541A. DOI: 10.1039/B508541A.
(10) Grimme, S.; Ehrlich, S.; Goerigk, L. Effect of the damping function in dispersion corrected density functional theory. *Journal of Computational Chemistry* **2011**, *32* (7), 1456-1465. DOI: https://doi.org/10.1002/jcc.21759.
(11) Johnson, B. G.; Fisch, M. J. An implementation of analytic second derivatives of the gradient-corrected density functional energy. *The Journal of Chemical Physics* **1994**, *100* (10), 7429-7442. DOI: 10.1063/1.466887 (acccessed 11/10/2023).
(12) Stratmann, R. E.; Burant, J. C.; Scuseria, G. E.; Frisch, M. J. Improving harmonic vibrational frequencies calculations in density functional theory. *The Journal of Chemical Physics* **1997**, *106* (24), 10175-10183. DOI: 10.1063/1.474047 (acccessed 11/10/2023).
(13) Marenich, A. V.; Cramer, C. J.; Truhlar, D. G. Universal Solvation Model Based on Solute Electron Density and on a Continuum Model of the Solvent Defined by the Bulk Dielectric Constant and Atomic Surface Tensions. *The Journal of Physical Chemistry B* **2009**, *113* (18), 6378-6396. DOI: 10.1021/jp810292n.
(14) Griffiths, D. J. *Introduction to Quantum Mechanics*; 1994.
(15) Nandy, A.; Chu, D. B. K.; Harper, D. R.; Duan, C.; Arunachalam, N.; Cytter, Y.; Kulik, H. J. Large-scale comparison of 3d and 4d transition metal complexes illuminates the reduced effect of exchange on second-row spin-state energetics. *Physical Chemistry Chemical Physics* **2020**, *22* (34), 19326-19341, 10.1039/D0CP02977G. DOI: 10.1039/D0CP02977G.
(16) Kühne, T. D.; Iannuzzi, M.; Del Ben, M.; Rybkin, V. V.; Seewald, P.; Stein, F.; Laino, T.; Khaliullin, R. Z.; Schütt, O.; Schiffmann, F. CP2K: An electronic structure and molecular dynamics software package-Quickstep: Efficient and accurate electronic structure calculations. *The Journal of Chemical Physics* **2020**, *152* (19), 194103.
(17) Godbout, N.; Salahub, D. R.; Andzelm, J.; Wimmer, E. Optimization of Gaussian-type basis sets for local spin density functional calculations. Part I. Boron through neon,

optimization technique and validation. *Canadian Journal of Chemistry* **1992**, *70* (2), 560-571. DOI: 10.1139/v92-079.
(18) Grimme, S.; Antony, J.; Ehrlich, S.; Krieg, H. A consistent and accurate ab initio parametrization of density functional dispersion correction (DFT-D) for the 94 elements H-Pu. *The Journal of Chemical Physics* **2010**, *132* (15). DOI: 10.1063/1.3382344 (acccessed 11/10/2023).
(19) Bussi, G.; Donadio, D.; Parrinello, M. Canonical sampling through velocity rescaling. *The Journal of Chemical Physics* **2007**, *126* (1), 014101. DOI: 10.1063/1.2408420.
(20) Goedecker, S.; Teter, M.; Hutter, J. Separable dual-space Gaussian pseudopotentials. *Physical Review B* **1996**, *54* (3), 1703-1710. DOI: 10.1103/physrevb.54.1703.
(21) Madsen, M. R.; Jakobsen, J. B.; Rønne, M. H.; Liang, H.; Hammershøj, H. C. D.; Nørby, P.; Pedersen, S. U.; Skrydstrup, T.; Daasbjerg, K. Evaluation of the Electrocatalytic Reduction of Carbon Dioxide using Rhenium and Ruthenium Bipyridine Catalysts Bearing Pendant Amines in the Secondary Coordination Sphere. *Organometallics* **2020**, *39* (9), 1480-1490. DOI: 10.1021/acs.organomet.9b00815.
